# Huntingtin contains an ubiquitin-binding domain and regulates lysosomal targeting of mitochondrial and RNA-binding proteins

**DOI:** 10.1073/pnas.2319091121

**Published:** 2024-07-29

**Authors:** Gianna M. Fote, Vinay V. Eapen, Ryan G. Lim, Clinton Yu, Lisa Salazar, Nicolette R. McClure, Jharrayne McKnight, Thai B. Nguyen, Marie C. Heath, Alice L. Lau, Mark A. Villamil, Ricardo Miramontes, Ian H. Kratter, Steven Finkbeiner, Jack C. Reidling, Joao A. Paulo, Peter Kaiser, Lan Huang, David E. Housman, Leslie M. Thompson, Joan S. Steffan

**Affiliations:** ^a^Department of Biological Chemistry, UC Irvine School of Medicine, Irvine, CA 92697; ^b^Department of Neurological Surgery, UC Irvine School of Medicine, Orange, CA 92868; ^c^Department of Cell Biology, Harvard Medical School, Boston, MA 02115; ^d^Casma Therapeutics, Cambridge, MA 02139; ^e^The University of California Irvine Institute for Memory Impairments and Neurological Disorders, University of California, Irvine, CA 92697; ^f^Department of Physiology and Biophysics, University of California, Irvine, CA 92697; ^g^Department of Psychiatry and Human Behavior, UC Irvine School of Medicine, Orange, CA 92868; ^h^Department of Neurobiology and Behavior, University of California, Irvine, CA 92697; ^i^Center for Systems and Therapeutics, Gladstone Institutes, San Francisco, CA 94158; ^j^Stanford Brain Stimulation Lab, Stanford, CA 94304; ^k^Department of Psychiatry and Behavioral Sciences, Stanford University, Stanford, CA 94304; ^l^Department of Physiology, University of California, San Francisco, CA 94158; ^m^Department of Neurology, University of California, San Francisco, CA 94158; ^n^Koch Institute for Integrative Cancer Research, The Massachusetts Institute of Technology, Cambridge, MA 02139; ^o^Center for Epigenetics and Metabolism, University of California, Irvine School of Medicine, University of California, Irvine, CA 92697

**Keywords:** Huntingtin, ubiquitin-binding domain, ubiquitin, autophagy, RNA-binding proteins

## Abstract

Understanding the normal function of the Huntingtin (HTT) protein is of significance in the design and implementation of therapeutic strategies for Huntington’s disease (HD). Expansion of the CAG repeat in the *HTT* gene, encoding an expanded polyglutamine (polyQ) repeat within the HTT protein, causes HD and may compromise HTT’s normal activity contributing to HD pathology. Here, we investigated the previously defined role of HTT in autophagy specifically through studying HTT’s association with ubiquitin. We find that HTT interacts directly with ubiquitin in vitro. Tandem affinity purification was used to identify ubiquitinated and ubiquitin-associated proteins that copurify with a HTT N-terminal fragment under basal conditions. Copurification is enhanced by HTT polyQ expansion and reduced by mimicking HTT serine 421 phosphorylation. The identified HTT-interacting proteins include RNA-binding proteins (RBPs) involved in mRNA translation, proteins enriched in stress granules, the nuclear proteome, the defective ribosomal products (DRiPs) proteome and the brain-derived autophagosomal proteome. To determine whether the proteins interacting with HTT are autophagic targets, HTT knockout (KO) cells and immunoprecipitation of lysosomes were used to investigate autophagy in the absence of HTT. HTT KO was associated with reduced abundance of mitochondrial proteins in the lysosome, indicating a potential compromise in basal mitophagy, and increased lysosomal abundance of RBPs which may result from compensatory up-regulation of starvation-induced macroautophagy. We suggest HTT is critical for appropriate basal clearance of mitochondrial proteins and RBPs, hence reduced HTT proteostatic function with mutation may contribute to the neuropathology of HD.

## Significance

Mutation compromising the normal function of the Huntingtin (HTT) protein is a possible contributor to pathology in Huntington’s disease (HD). We show here that HTT knockout alters lysosomal trafficking of mitochondrial and RNA-binding proteins (RBPs). We demonstrate that the HTT protein can directly bind ubiquitin through a ubiquitin-binding domain (UBD) that interacts with ubiquitinated or ubiquitin-associated RBPs, likely impacting cellular proteostasis, suggesting a mechanism in which HTT scaffolds these proteins for degradation.

Huntington’s disease (HD) is a devastating neurodegenerative disorder caused by a CAG repeat expansion in the Huntingtin gene (*HTT*) and corresponding polyglutamine (polyQ) expansion in the amino-terminal domain of the HTT protein, resulting in age-related loss of medium spiny neurons in the striatum and neuropathological alterations in the cortex (1). Although the disease gene was cloned 30 years ago, there are still no treatments for patients that alter the course of the disease. As therapeutic strategies are developed for HD, it is critical to understand the normal functions of HTT that may in turn be impacted by the presence of the mutation. HTT is ubiquitously expressed, essential for human development, and mediates a number of intracellular functions, including vesicle recycling, endosomal trafficking, regulation of transcription and translation, and coordination of cell division (2–4). We and others previously determined that one of the normal functions of the wild-type (wt) full-length HTT protein is that of a scaffold for selective autophagy (5–8). We found that HTT contains domains similar to yeast autophagy proteins Atg23, Vac8, and Atg11 important for the yeast cytoplasm to vacuole targeting (CVT) pathway, and determined that HTT could bind autophagic receptor proteins and core autophagy machinery as shown for yeast Atg11 (5, 7). Atg23 is a vesicle-tethering protein involved in yeast macroautophagic trafficking (9, 10), and Vac8 plays a role in yeast microautophagy (11); this suggests that HTT may function in both macroautophagy and microautophagy in mammalian cells. Consistent with reduced autophagic function associated with aging and inflammation (12), we observed that HTT levels decline with age in the striatum and cortex, tissues that degenerate in HD, and that the proinflammatory kinase IKKbeta may activate HTT’s autophagic function through phosphorylation of HTT serine 13 in vivo (8, 13, 14). Loss of HTT’s autophagic function with mutation and aging may contribute to proteostatic stress in HD; up-regulation of macroautophagy, chaperone-mediated autophagy (CMA) and proteasome activity have all been observed in HD models (14–18).

The trafficking of autophagosomes, endosomes, and lysosomes may be important for HTT’s autophagic function (3, 19, 20). HTT controls autophagosome dynamics in neurons, and retrograde axonal transport of autophagosomes is impaired in HD knock-in mice correlating with inefficient degradation of engulfed mitochondrial fragments (19). HTT-mediated vesicular transport in neurons can either be anterograde or retrograde, depending on whether HTT interacts with dynein or kinesin proteins, which propel vesicles along microtubules (21). The ability to switch motor protein complexes and directions can be mediated by phosphorylation of HTT serine 421 (S421). HTT and dynein-mediated retrograde transport of vesicles toward the soma, the location of most mature lysosomes, is enhanced by dephosphorylation of S421 by the phosphatase calcineurin (22). Conversely, phosphorylation of HTT S421 by prosurvival kinase Akt or by the serum- and glucocorticoid-induced kinase S6K recruits kinesin-1 to the dynactin complex on vesicles to promote anterograde transport (3, 21).

Ubiquitin-binding domains are a common feature of proteostasis receptor proteins targeting ubiquitinated proteins for degradation by the proteasome and lysosome, but can also mediate nonproteolytic mechanisms including endocytosis, vesicular trafficking, cell-cycle control, stress response, DNA repair, growth-factor signaling, transcription, and gene silencing (23, 24). Wt HTT N-terminal fragment copurifies noncovalently with ubiquitin, suggesting a direct interaction between HTT and ubiquitin, potentially modulated by HTT phosphorylation (25). Further, one of the early neuropathologic findings in HD mice and human brain following identification of the disease gene was the presence of HTT nuclear inclusions that colocalized with ubiquitin (26, 27).

Here, we defined ubiquitin-binding with a normal repeat-containing HTT N-terminal fragment to further study HTT protein function and to understand how loss of these activities might specifically disrupt protein homeostasis in HD. We investigated autophagic targets of HTT by examining its ubiquitinated binding partners and interrogating the contents of lysosomes following HTT knockout (KO). With HTT KO, mitochondrial protein targeting to the lysosome is impaired, mitochondrial oxidative stress is enhanced and mitochondrial health is reduced, supporting that HTT scaffolds a mitophagic pathway. However, macroautophagy and lysosomal clearance of RNA-binding proteins (RBPs) are up-regulated upon HTT KO. Tandem purification of tagged wt N-terminal HTT fragment and ubiquitin from transiently transfected neuronal precursor St14A cells was carried out to investigate HTT’s autophagic targets. We identified a group of ubiquitinated or ubiquitin-associated proteins that significantly and reproducibly copurified with HTT. Copurification is enhanced with HTT polyQ expansion but reduced by mimicking HTT phosphorylation at serine 421. The ubiquitinated proteins that interact with HTT include known targets of macroautophagy and CMA (28–31). The most significant group of potentially ubiquitinated proteins that copurified with HTT are RBPs involved in mRNA translation. Finally, HTT KO alters abundance of stress granules, which are composed of many of the RBPs that copurify with HTT. Therefore, HTT interactions with ubiquitinated autophagic cargo proteins may be mediated by an ubiquitin-binding domain (UBD) within HTT residues 235 to 367 that may facilitate HTT’s function in selective autophagy.

## Results

### HTT KO Modulates Proteostasis.

**Fig. 1.**
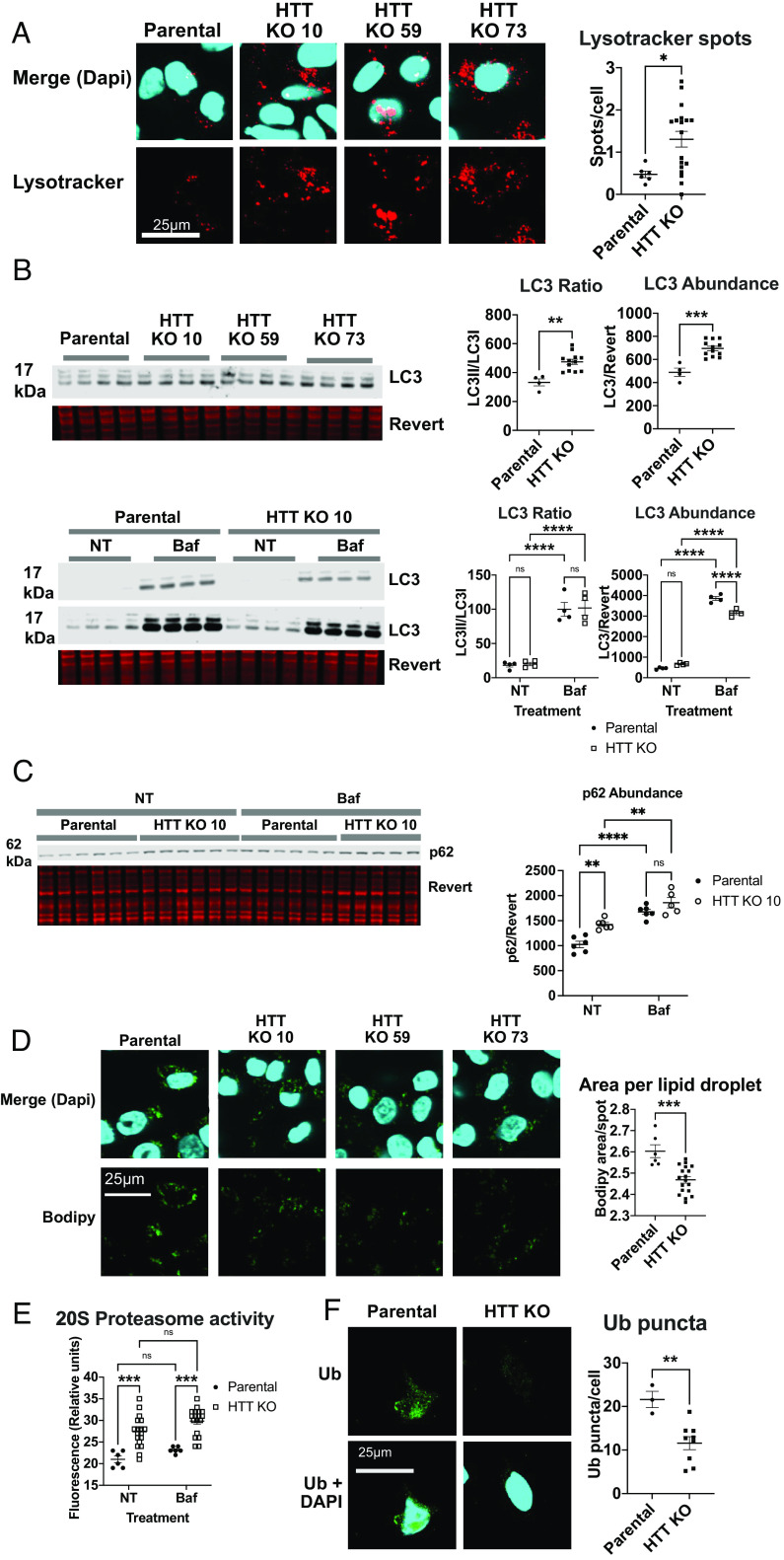
Proteostasis is disrupted in 8988T TMEM192-HA cells by HTT KO. (*A*) Lysotracker red staining was performed on six wells of parental and HTT KO cell lines, and three images were averaged per well. Spots were quantified using Imaris software and compared using a two-tailed *t* test. (*B*) LC3 abundance and ratio of LC3II to LC3I were evaluated by western blot with or without 4 h of 50 nM Baf treatment. Whole protein levels were quantified using revert protein stain from LI-COR Biosciences. (*C*) Abundance of autophagic receptor p62 was evaluated by western blot with and without Baf treatment. (*D*) Lipid droplet staining was performed using Bodipy following overnight oleic acid (200 nM) treatment on six wells of each cell line. Three images were averaged per well. For all statistical analyses, three HTT KO cell lines were grouped. (*E*) 20S proteasome activity was analyzed using a fluorescence-based assay with and without Baf treatment at 50 nM for 4 h using HTT KO clone 10. (*F*) Ubiquitin puncta were quantified using Imaris software from HTT KO clone 10. Confocal microscopy was used to take three 20× images per well, three wells per cell line. In all statistical analyses, replicates from two or three HTT KO clonal lines were analyzed together as biological replicates.

Proteostasis can be maintained by several complementary pathways, including macroautophagy, microautophagy, CMA, and the proteasome (32). Given HTT’s previously defined role as an autophagic scaffold protein (5, 6), we investigated the contribution of HTT to proteostasis in human cells. Homozygous HTT KO cell lines were created using CRISPR-mediated genome editing of a pancreatic adenocarcinoma cell line PATU8988T, which stably expresses a tagged lysosomal protein, TMEM192-HA (*SI Appendix*, Fig. S1). We compared the parental and HTT KO 8988T cell lines for evidence of macroautophagic dysregulation in the absence of HTT. First, we observed a significant up-regulation of lysosome numbers and size with HTT KO using lysotracker staining (Fig. 1*A*). Second, we examined autophagic flux by analyzing the ratio of LC3II, the lipidated form when conjugated to autophagosome membranes (32) to LC3I (nonlipidated form) levels in 8988T whole-cell lysates. LC3 II/I ratio and LC3 abundance were both elevated in HTT KO cells (Fig. 1*B*), indicating that macroautophagy may be up-regulated and autophagosome formation increased in HTT KO cells. Since LC3 levels can be dependent on the degree of lysosomal degradation as well as autophagic activation, the lysosomal inhibitor Bafilomycin A1 (Baf) was used to assess autophagic flux. With the addition of Baf, LC3 accumulates significantly in both cell lines, suggesting that autophagic turnover is occurring, and the increased LC3II/LC3I ratio in HTT KO cells may demonstrate high macroautophagic activity. Additionally, HTT KO elevated levels of p62, an autophagy receptor that is turned over with macroautophagy. p62 levels are further increased by Baf, suggesting that autophagic turnover of this receptor is active in both cell lines (Fig. 1*C*). We also observed reduced numbers of lipid droplets with HTT KO, consistent with the possibility of enhanced macroautophagic lipid droplet degradation (macrolipophagy) (33), which can free fatty acids for further autophagosome formation (Fig. 1*D*).

Macroautophagy and the proteasome can work either in a compensatory fashion when one is impaired, or synergistically to overcome proteostatic disruption (34). We observed increased 20S proteasome activity (Fig. 1*E*) and reduced ubiquitin puncta in HTT KO cells (Fig. 1*F*). Both macroautophagy and the proteasome appear to be up-regulated in HTT KO cells. Finally, we examined levels of the CMA receptor LAMP2A, which is significantly more abundant in HTT KO cells (*SI Appendix*, Fig. S2*A*). Thus, multiple autophagic pathways and the proteasome are dysregulated with HTT KO, consistent with a requirement for HTT in proteostasis. Increased macroautophagy and proteasome activity suggest possible compensation for loss of HTT proteostatic function, leading to the question whether HTT might selectively target proteins to the lysosome for alternative autophagy pathways.

### HTT KO Alters Cellular Lysosomal Targeting of Mitochondrial Proteins and RBPs.

To investigate whether HTT may selectively target proteins to the lysosome, we analyzed the contents of immunoprecipitated intact lysosomes in the PATU8988T HTT KO cells (*SI Appendix*, Fig. S2*B*). The tagged lysosomal protein, TMEM192-HA, allowed us to immunoprecipitate (IP) whole, intact lysosomes for mass spectrometry analysis. To preserve lysosomal contents, cells were treated with the lysosomal inhibitor Baf for four hours and gently lysed. An 8988T cell line not expressing TMEM192-HA was included as a negative control for lysosomal enrichment. Western blotting was used to verify HTT KO (HTT KO line 10 shown). Successful lysosomal IP (LysoIP) was demonstrated by enrichment of LAMP1 in the eluate of parental and HTT KO cell lines, but not the no-tag control line (*SI Appendix*, Fig. S2*B*). The lysosomal proteome of three cell lines, the wt parental cell line (Parental) and two HTT KO cell lines (HTT KO 10 and HTT KO 59), were analyzed by tandem-mass-tag-based quantitative mass spectrometry. Additionally, we previously found an activation of macroautophagy with HTT KO in 8988T cells (Fig. 1). To assess the effect of macroautophagy activation on lysosomal contents in cells containing HTT, we starved 8988T cells with HBSS to activate starvation-induced macroautophagy and then evaluated lysosomal contents. LysoIP experiments had at least two replicates in each experiment for each cell line.

**Fig. 2.**
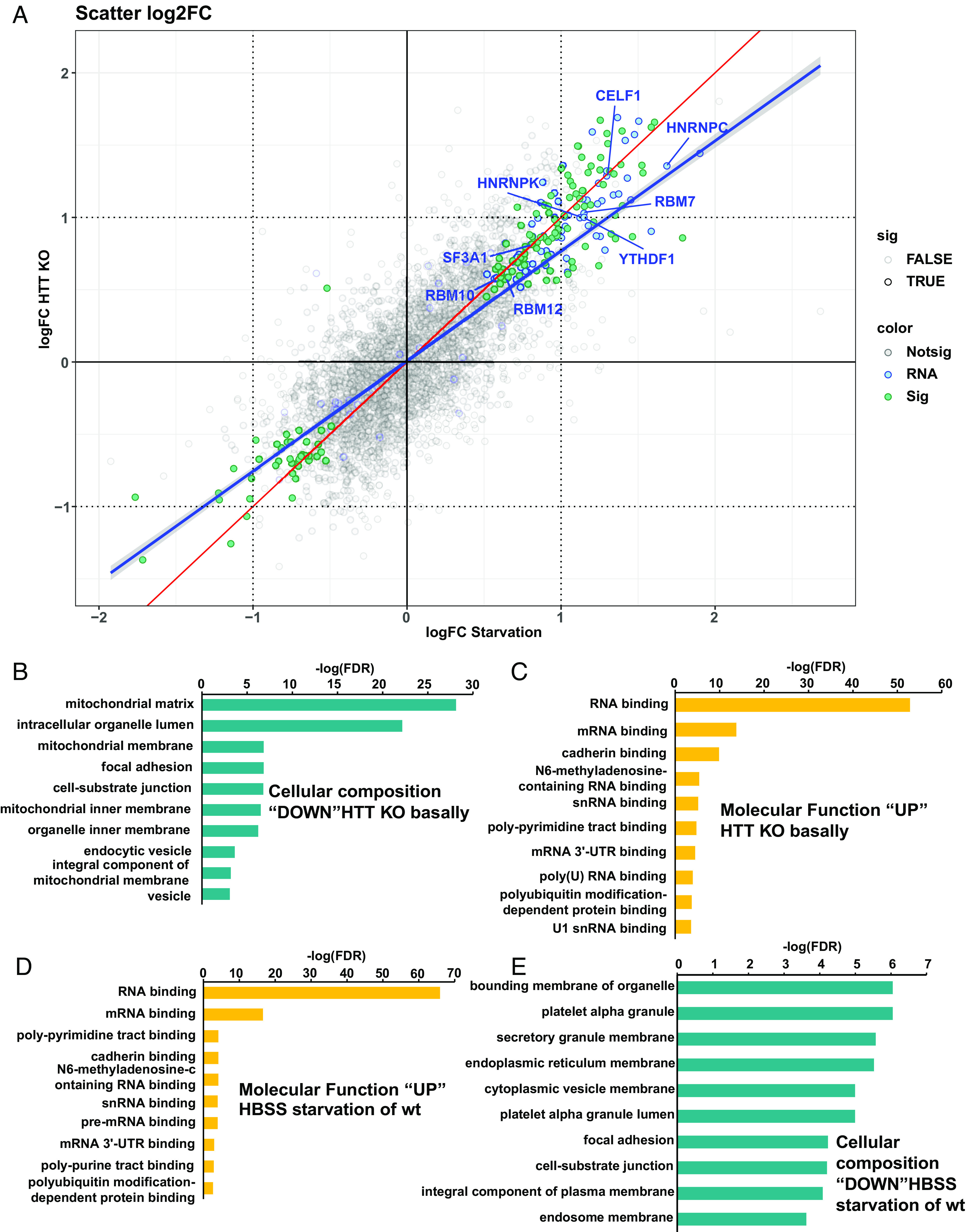
HTT KO alters the contents of lysosomes. LysoIP was performed in 8988T cells expressing TMEM192-HA using magnetic HA pulldown beads. All cells were treated with Baf for 4 h prior to harvest in order to preserve lysosomal proteins for analysis. Proteomic contents of purified lysosomes were analyzed by mass spectrometry. Scatterplot of log2 fold changes comparing HTT KO to WT starvation-induced macroautophagy (turquoise = significant in both lists, blue = RNA- or mRNA-binding protein, red line = perfect correlation, blue line = LOESS curve fit, labeled proteins are exemplary). (*A*). Gene ontology pathways significantly altered by HTT KO were identified using Enrichr. Threshold for significant enrichment was FDR < 0.1. (*B*) Cellular composition enrichment of proteins that were decreased in the lysosomes of HTT KO cells compared to parental cells. (*C*) Molecular function enrichment of proteins that were increased in the lysosomes of HTT KO cells compared to parental cells. (*D*) Molecular function enrichment of proteins that were increased in the lysosomes of parental cells with HBSS treatment for 4 h prior to harvest, for stimulation of macroautophagy. (*E*) Cellular composition enrichment of proteins that were decreased in the lysosomes of parental cells with HBSS treatment for 4 h prior to harvest did not include mitochondrial proteins, unlike what we observed with HTT KO basally.

After QC assessment, normalized protein quantification data were used for statistical analysis (see **SI Appendix*, *Materials and Methods**) to identify differentially abundant proteins. Comparing HTT KO lysosomal cargo against wt cargo we identified 575 differential proteins (FDR < 0.1) and comparing wt starvation-induced macroautophagy lysosomal cargo to wt basal cargo, we identified 429 differential proteins (*SI Appendix*, Fig. S3). To understand whether HTT KO was impacting similar lysosomal cargo as wt starvation-induced macroautophagy, we compared both lists and identified 204 overlapping proteins, a significant enrichment over chance calculated by exact hypergeometric probability (*P* < 1.632e−197). We next assessed whether protein changes in both conditions were also affecting proteins in the same direction of change; Fig. 2*A* shows a scatterplot of log2 fold changes of all proteins identified in the mass spectrometry experiment between both conditions. The plot shows a clear positive correlation between HTT KO and starvation (blue line, LOESS curve fit) suggesting that not only does HTT KO impact similar proteins as starvation, but also affects them in a similar manner of abundance directionality. We noticed a large number of proteins involved in RNA binding in the up-regulated lists of both conditions (proteins significant in both lists are highlighted in turquoise and genes found in the GO term RNA-binding protein or mRNA binding are highlighted in blue). We speculated that there was an enrichment of RNA-binding protein in these conditions. To confirm this, we ran gene ontology analysis on the sets of proteins that were underrepresented (“DOWN”) or overrepresented (“UP”) in the lysosomal proteome from HTT KO cells. Mitochondrial proteins were significantly “DOWN”, presumably inhibited from entering the lysosome, in HTT KO cells compared with parental lines (Fig. 2*B*), while the molecular function of proteins with significantly increased abundance in the lysosome with HTT KO was enriched for RBPs (Fig. 2*C*). As we speculated from the protein list comparisons, assessing the effects of starvation-induced macroautophagy on wt cells showed a similar enrichment of RBPs (Fig. 2*D*). 56% of the proteins identified as up-regulated by serum starvation in Fig. 2*D* overlapped with proteins with increased lysosomal abundance in HTT KO cells, and the most significant molecular function categories among these protein sets were RNA binding and mRNA binding (Fig. 2 *C* and *D* and Datasets S1 and S2). We noted that the cellular composition of proteins “DOWN” in the lysosome with serum starvation was not mitochondrial (Fig. 2*E*), unlike what we observed here with HTT KO basally (Fig. 2*B*). Thus, HTT may contribute to basal mitophagy, and HTT KO may activate starvation-induced macroautophagy, increasing the lysosomal trafficking of RBPs.

### HTT Copurifies Noncovalently With Ubiquitinated or Ubiquitin-Associated RBPs.

**Fig. 3.**
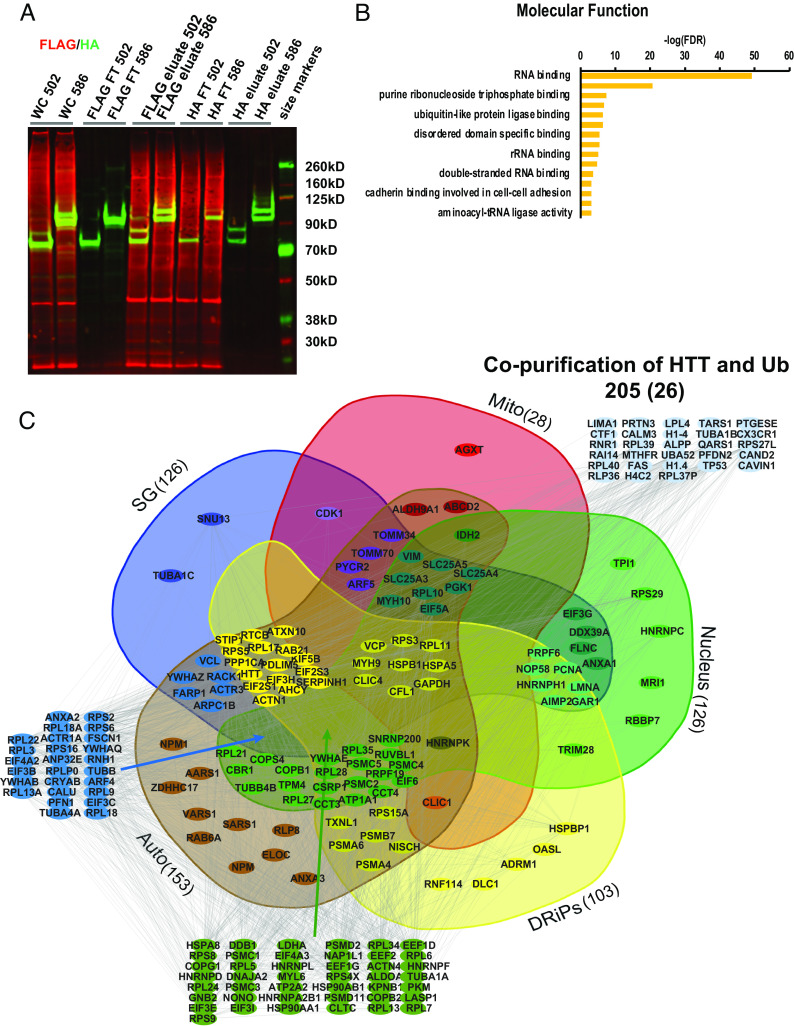
HTT copurifies with potentially ubiquitinated RNA-binding proteins. 17Q-502-HIS-HA-HA-HIS and FLAG-ubiquitin were coexpressed in St14A cells, crosslinked with formaldehyde, tandem copurified, and subjected to mass spectrometry analysis. (*A*) Plasmids encoding 17Q N-terminal 502 or 586 amino acid HTT fragments C-terminally tagged with HIS-HA-HA-HIS were transfected into St14A cells together with FLAG-ubiquitin. Cells were subjected to crosslinking with formaldehyde 2 d post-transfection, then lysed. Lysates were incubated overnight with an anti-FLAG matrix, washed then eluted with FLAG peptide, then incubated overnight with an anti-HA matrix, washed, and eluted with glycine pH 2. Samples from each copurification step were subjected to western analysis with anti-FLAG MAB (red) and polyclonal anti-HA antibody (green). FT denotes flow through of proteins that did not bind matrix. The final HA eluate shows bands the size of unmodified HTT fragment (green), and also a monoubiquitinated species (both red and green), together with copurifying ubiquitinated proteins (red). (*B*) Molecular function analysis of the ubiquitinated proteins associated noncovalently with HTT showed them to be most significantly RBPs. (*C*) 205 proteins copurified significantly above vector control in at least 2 of 3 tandem purifications. These proteins were found in protein databases for the nucleus (61%), stress granules (SG, 61%), defective ribosomal products (DRiPs, 50%), brain-derived autophagosomes (auto, 75%), and mitochondria (mito, 14%).

As an autophagic scaffold, HTT may interact with ubiquitinated proteins bound for lysosomal degradation (5–8, 13, 35). We previously found that transiently transfecting a mouse/human hybrid wt 17Q-HTT 480 amino acid (aa) fragment coimmunoprecipitated noncovalently with HA-tagged ubiquitinated proteins purified from St14A immortalized striatal precursor cells (25), suggesting this HTT fragment contained an ubiquitin-binding domain. To study this interaction in more depth, we created plasmids expressing human wt 17Q-502aa and 586aa C-terminally tagged with HIS-HA-HA-HIS, coexpressed these in St14A cells with FLAG-ubiquitin, and used western analysis with anti-HA/anti-FLAG antibodies and LI-COR detection. We confirmed the noncovalent coimmunoprecipitation of both human HTT fragments with ubiquitinated proteins, including bands that are not size-shifted and therefore represent a noncovalent interaction with ubiquitin rather than a covalent ubiquitination. We chose the 502 fragment (the equivalent of the mouse/human hybrid 480 aa fragment) for further studies as it was sufficient in length for full interaction (Fig. 3*A*).

We investigated the identity of the ubiquitinated/ubiquitin-associated proteins that this HTT fragment copurifies with in St14A cells using mass spectrometry and a tandem purification technique we previously developed (36). 17Q-HTT 502-HIS-HA-HA-HIS in pcDNA3.1, or pcDNA3.1 vector control with FLAG-ubiquitin were transiently transfected, crosslinked with formaldehyde, and cells lysed. Lysates were run on an anti-FLAG M2 column, washed, eluted with FLAG peptide, run on an anti-HA column, washed, and eluted with glycine, and then subjected to mass spectrometry analysis (as in Fig. 3*A*). In three separate purifications using 502 fragment or vector control, we found 205 proteins that significantly copurified with HTT above vector control in at least 2 of 3 runs. These ubiquitinated proteins/ubiquitin-associated proteins bound to HTT were most significantly enriched in pathways involving RNA-binding in mRNA translation (Fig. 3*B* and Dataset S3). These interacting proteins also include proteins identified in basal autophagosomes isolated from the mouse brain (28), and those that accumulate with Lamp2A KO-mediated loss of CMA (29–31), suggesting that these interacting proteins may be autophagic targets. We identified proteins previously found in the mitochondrial proteome compiled by the University of Cambridge MRC Mitochondrial Biology Unit and by the Broad Institute (MitoCarta3.0) and identified RBPs found in stress granules, the nucleus, and in defective DRiPs (37–39) (Fig. 3*C* amd Dataset S4).

### HTT Amino Acids 235-367 Directly Interact with Ubiquitin In Vitro.

Although many autophagy proteins are known to contain ubiquitin-binding domains (UBDs), a UBD within HTT has not previously been described. To map the direct noncovalent interaction of HTT with ubiquitin, we generated GST-HTT fusion proteins to determine which amino acids were required to directly bind ubiquitin in vitro. Using GST pull-down analyses with the ubiquitin-binding UBA domain of p62 fused to GST as a positive control and GST alone as a negative control, and recombinant purified M1-linear hexa-ubiquitin, we identified the area of direct interaction with ubiquitin as between HTT amino acids 235-367 (Figs. S4 and S5*A*). This domain preferred interaction with K48 to K63 ubiquitin chains, unlike the p62-UBA, which preferred K63 over K48 chains (*SI Appendix*, Fig. S4*D*). Ubiquitin isoleucine 44 (I44) is a common residue required for most ubiquitin-binding domain interactions and mutation to alanine can generally disrupt this binding (35). Phosphorylation of serine 65 (S65) of ubiquitin enhances recruitment of the ubiquitin-binding region of Parkin activating its function in mitophagy; mimicking ubiquitin phosphorylation by changing S65 to glutamic acid can enhance this interaction (40, 41), therefore we assessed binding of HTT to both I44A and S65E ubiquitin mutants in addition to wt ubiquitin. HTT 235-367 fragment interacted directly in vitro with wt M1-linked linear hexa-ubiquitin, more strongly with I44A M1-linked linear hexa-ubiquitin, and less well with phosphomimetic S65E M1-linked linear hexa-ubiquitin (*SI Appendix*, Figs. S4 and S5*B*). In contrast, the GST-p62 UBA did not bind I44A, but did bind wt ubiquitin. GST-p62 UBA also bound S65E ubiquitin, although significantly less strongly than wt (*SI Appendix*, Fig. S5*C*). Our data demonstrates that the UBD of HTT is different from the UBA of p62 and may have more in common with Ubiquitin-Binding Motif (UBM) domains previously shown to interact with I44A ubiquitin (42).

### Point Mutations Destabilize the Interaction of HTT 235-367 with Ubiquitin In Vitro.

**Fig. 4.**
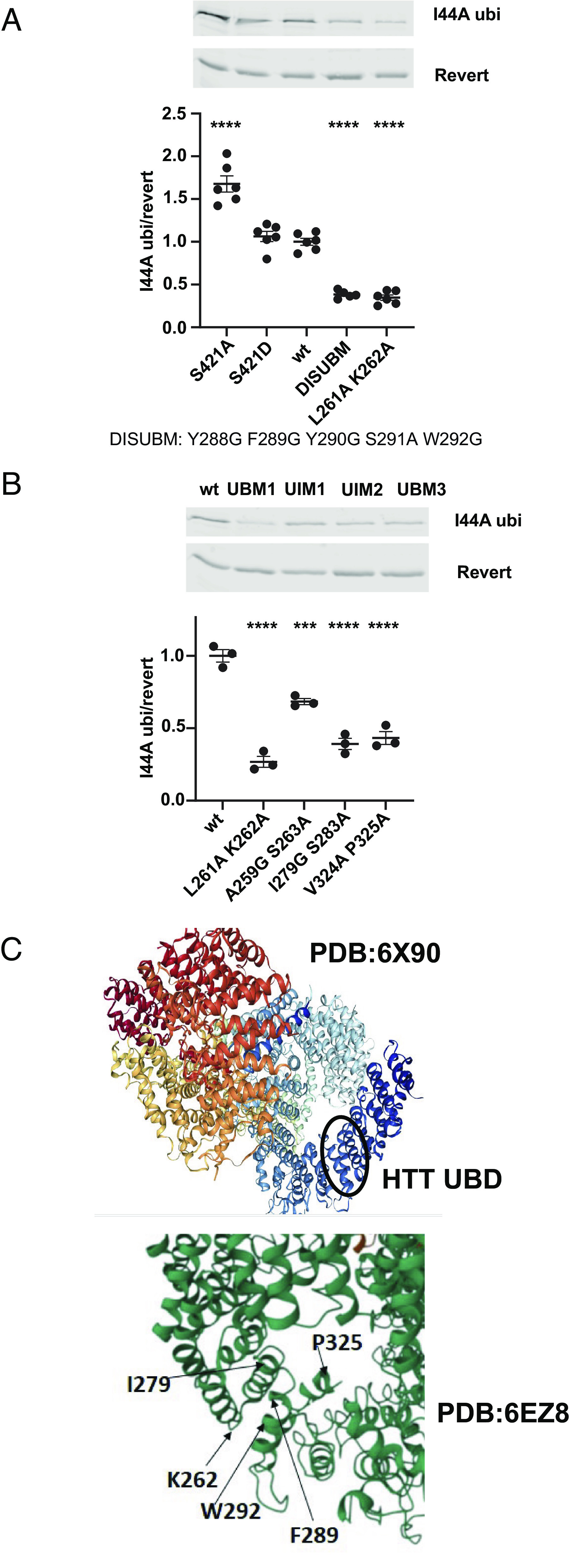
Mutation of amino acids within HTT’s 235-367 amino acid ubiquitin-binding domain destabilizes the in vitro interaction with I44A ubiquitin. (*A*, *B*) HTT GST-235-502 fragment was incubated in vitro with I44A ubiquitin. DISUBM (Y288G F289G Y290G S291A W292G) and UBM1 (L261A K262A), UIM1 (A259G S263A), UIM2 (I279G S283A), and UBM3 (V324A P325A) mutations reduced the in vitro interaction between I44A ubiquitin and HTT, while S421A enhanced the interaction as analyzed by One-Way ANOVA with Bonferroni’s multiple comparisons test and compared with wt control. (*C*) Cryo-EM structure from PDB:6X90 (46) or 6EZ8 (47) demonstrates the domain (residues 261 to 325, black circle) and amino acids involved in the in vitro HTT interaction with I44A ubiquitin.

As I44 is an ubiquitin residue required for interaction with most UBDs (23), we evaluated HTT for similarities with ubiquitin-binding motif (UBM)-containing proteins and disordered UBM (DisUBM)-containing proteins that do not require ubiquitin I44 for interaction. UBMs and DisUBMs can interact directly with the I44A form of ubiquitin unlike most other UBDs (42, 43). We visually scanned our in vitro UBD amino acid sequence between HTT amino acids 235-367 and found three potential UBM domains (44), two potential ubiquitin-interacting)motifs (UIMs) (45), and one DisUBM consensus sequence (43) (*SI Appendix*, Fig. S6). Each of these six potential UBDs were mutated and analyzed using the in vitro GST-pull down assay with M1-linked linear I44A hexa-ubiquitin (Fig. 4 *A* and *B*). The largest reduction in I44A ubiquitin binding was observed for mutants L261A K262A (UBM1), Y288G F289G Y290G S291A W292G (DISUBM), I279G S283A (UIM2), and V324A P325A (UBM3) (Fig. 4 *A* and *B*). All of these mutations are found in a domain with three alpha helices (Fig. 4*C* and *SI Appendix*, Figs. S6 and S7) defined in the Cryo-EM structure of HTT: PDB:6X90 (46) and PDB:6EZ8 (47). One mutant, V302A P303A (UBM2), enhanced in vitro binding to I44A ubiquitin, as did S421A, which lies outside the HTT ubiquitin-binding domain (Fig. 4*A* and *SI Appendix*, Fig. S8). V302A P303A is adjacent to three acidic residues D305, E306, and D307 which could be relevant to function of a HTT DisUBM domain (*SI Appendix*, Figs. S6 and S8) (43) and enhance the ability of these residues to influence ubiquitin-binding of the DisUBM.

### HTT Fragment Noncovalent Interaction with Ubiquitin is Modulated by Posttranslational Modification.

We previously determined that mimicking phosphorylation of S421 using an S421D mutation increased proteasomal clearance of human/mouse hybrid 480 amino acid fragment (25). Here, we also observe that S421D human 17Q-502-HIS-HA-HA-HIS fragment levels were significantly reduced compared with wt in ST14A cells (*SI Appendix*, Fig. S9*A*). Therefore, we examined whether S421 could modulate copurification with ubiquitinated proteins. 17Q-1-502-HIS-HA-HA-HIS HTT wt and mutant fragments were coexpressed with FLAG-ubiquitin in St14A cells. Total HTT was pulled down with anti-HA monoclonal antibody, and separately ubiquitinated proteins were precipitated with anti-FLAG antibody, using equal micrograms of lysate. The relative coimmunoprecipitation of HTT with ubiquitin was quantitated comparing levels of anti-HA immunoreactivity coming down with anti-FLAG (FLAG-ubiquitinated proteins) vs. levels of anti-HA immunoreactivity coming down with anti-HA (total HTT). The immunoblots were detected with rabbit polyclonal anti-HA antibody. The S421D phosphomimetic mutant coimmunoprecipitated with FLAG-ubiquitin significantly less well than wt 502 fragment, which copurified with ubiquitin similarly to phosphoresistant S421A 502 fragment (*SI Appendix*, Fig. S9*B*). We created 10 additional mutants in this region: R418A, S421A S434A, V423P, L425V, L425A, G428V, G429V, G430V, L437P, and A452P (*SI Appendix*, Fig. S10*A*). Significantly reduced abundance of phosphomimetic S421D as well as mutants L425V, G428V, G429V, and A452P relative to wt 502 fragment was observed, suggesting amino acids adjacent to S421 can also regulate HTT stability. In addition to S421D, mutants L425V, G429V, and A452P coimmunoprecipitated with FLAG-ubiquitin significantly less well than wt 502 fragment (*SI Appendix*, Fig. S10*B*), however, there remained some binding.

We next investigated whether S421, while not required for interaction, might still modulate binding in vitro. M1-linked linear I44A hexa-ubiquitin was used to evaluate the HTT/ubiquitin interaction as this showed the most robust interaction with HTT fragment in our in vitro assay. S421A, which coimmunoprecipitated with FLAG-ubiquitin at similar levels to wt (*SI Appendix*, Fig. S9*B*), had enhanced in vitro interaction with I44A ubiquitin (*SI Appendix*, Fig. S9*C*). The reduced co-IP observed in cells in S421D was not observed for in vitro binding with I44A ubiquitin and in fact was slightly enhanced. G429V, which had reduced abundance and coimmunoprecipitation with ubiquitin in cells (*SI Appendix*, Fig. S9AB), had enhanced interaction in vitro with I44A ubiquitin (*SI Appendix*, Fig. S9*C*).

Here, using phosphomimetic and phosphoresistant constructs, we observe that the phosphorylation status of S421 can influence HTT’s interaction with ubiquitin in vitro and in cells, as S421A enhanced in vitro binding to I44A ubiquitin and S421D reduced coimmunoprecipitation with ubiquitin in cell culture. The observed in vitro interaction with ubiquitin and coimmunoprecipitation from cells do not necessarily mirror each other, suggesting the involvement of other factors in cells. While HTT amino acids 235-367 are involved in the direct interaction with ubiquitin in GST pull-down assays in vitro, or ubiquitinated proteins in cell culture, amino acids 368-502 may contain a regulatory domain that can modulate the direct interaction.

Two of the mutants within the domain of direct ubiquitin interaction (amino acids 235-367), L261A K262A and DISUBM, reduced in vitro binding to I44A ubiquitin most robustly, but still bound weakly (Fig. 4*A*). To evaluate the effect of these mutations on ubiquitin binding in cells, we generated the same mutations in the context of 17Q-HTT 502-HIS-HA-HA-HIS. St14A cells were cotransfected with the control and mutant constructs together with FLAG-ubiquitin and coimmunoprecipitated as in Fig. 3. Both mutants showed significantly enhanced coimmunoprecipitation with FLAG-ubiquitin compared with control but had normal abundance in cells on HA-IP (*SI Appendix*, Fig. S9*D*), unlike S421D or G429V mutations which had lower abundance and reduced ubiquitin coimmunoprecipitation (*SI Appendix*, Fig. S9 *A*, *B*).

Our data suggest that residues within HTT 235-367 function to bind ubiquitin similar to DisUBM motifs which have a low affinity for ubiquitin that is moldable by adjacent disordered amino acids chains allowing for an enhanced interaction surface with ubiquitin (43), and that the intrinsically disordered domain between 368 and 502 (*SI Appendix*, Fig. S6*D*) (47, 48) may regulate this binding function in vitro and in cells. While a direct in vitro interaction is readily defined, the interaction between HTT and ubiquitin in cells may be influenced by other cellular factors, including signaling pathways and calcineurin, leading to post-translational modification of cellular proteins.

### HTT Fragment Noncovalent Interaction with Ubiquitinated Proteins Is Modulated by CAG Repeat Expansion.

**Fig. 5.**
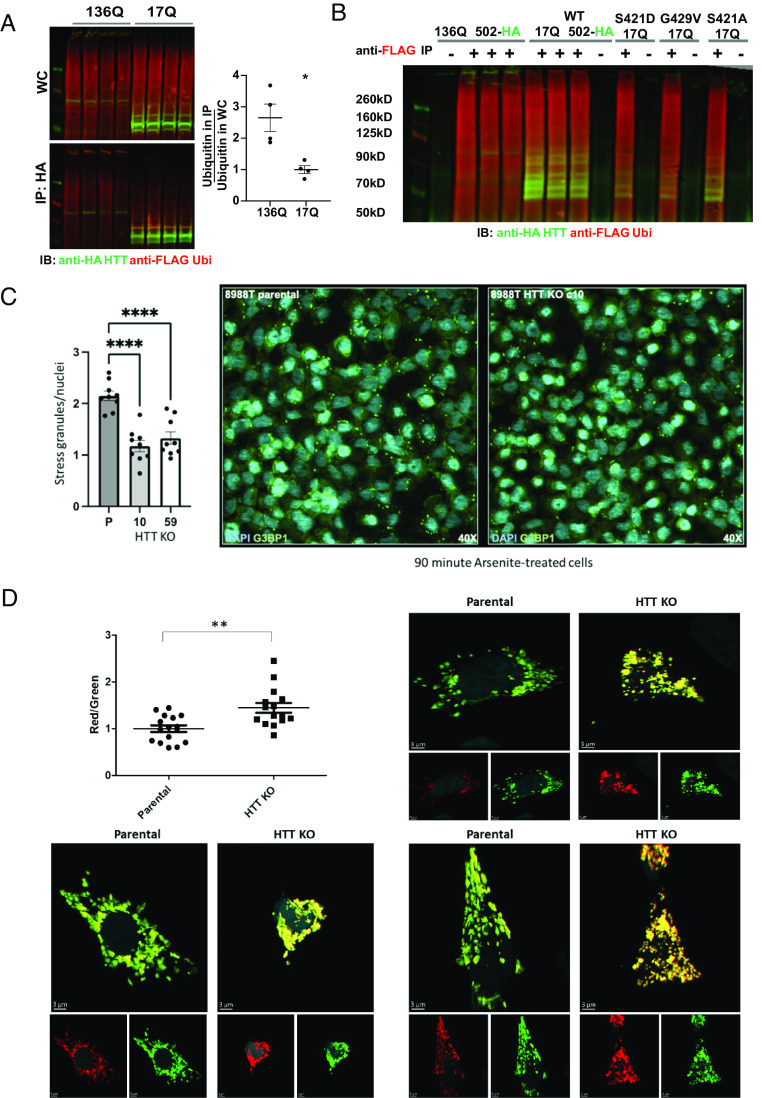
Higher levels of ubiquitinated proteins coimmunoprecipitate with mutant HTT fragment compared with wt control, and HTT KO alters RBP-containing stress granule clearance and mitochondrial health. (*A*) St14A cells were cotransfected with FLAG-ubiquitin and with 17Q-502-HIS-HA-HA-HIS (wt) vs. 136Q-502-HIS-HA-HA-HIS HTT (mutant) fragment. Whole-cell (WC) lysate was used for anti-HA MAB immunoprecipitation. Western analysis demonstrates that ubiquitinated proteins coimmunoprecipitate with wt HTT fragment, and the expansion of the polyQ repeat significantly enhances HTT binding to ubiquitinated proteins. Statistical analysis was done with an unpaired *t* test (*P* = 0.0108). (*B*) FLAG-ubiquitin does not stick nonspecifically to the protein G dynabeads used for immunoprecipitation. Whole-cell lysates of St14A cells expressing FLAG-ubiquitin together with either 136Q- or 17Q- wt, S421D, G429V, or S421A 502 HTT-HIS-HA-HA-HIS fragment were subjected to immunoprecipitation with mouse anti-FLAG antibody (+) or zero (−) antibody control. While FLAG-ubiquitin was easily purified with this anti-FLAG immunoprecipitation, FLAG-ubiquitin did not bind nonspecifically to protein G dynabeads used to capture the antibody. Western immunoblot (IB) was incubated with mouse anti-FLAG and with rabbit anti-HA antibodies for detection. (*C*) HTT KO reduces the number of arsenite-induced G3BP1-positive stress granules in 8988T TMEM192-HA cells. Cells were treated with acute sodium arsenite stress (125 μm NaAsO_2_) for 90 min, then fixed and stained with G3BP1 (green) and Hoechst (cyan). For each condition, quantitation of cells with G3BP1-positive stress granules (SGs) and Hoechst markers were captured. SG and nuclei quantification were completed using Cell Profiler. A parameter was created which normalized the SG count to nuclei in a single image to help control for differences in cellular confluency attributed to stressors. Data from the parental (P) and each HTT KO clone (clone 10 and clone 59) were statistically analyzed using a One-Way ANOVA (F(2, 24) = 23.34, *P* < 0.0001; n = 9, mean ± SEM; ****P < 0.0001). (*D*) HTT KO significantly increases the MitoTimer red/green ratio indicative of increased mitochondrial oxidative stress and reduced mitochondrial health. 8988T parental or clone 10 HTT KO cells were transfected with pMitoTimer and images were analyzed by Imaris software using the surface module to measure the sum of green intensity and the sum of red intensity of each cell. Statistical analysis was done by an unpaired *t* test.

To determine whether CAG repeat expansion altered the interaction of HTT with ubiquitin and ubiquitinated proteins, we constructed HIS-HA-HA-HIS-tagged human mutant 136Q-502 fragment and found that it significantly coimmunoprecipitated higher levels of ubiquitinated proteins relative to levels observed with the 17Q-502 fragment. This was the case even though the abundance of soluble 136Q-502 was much lower than 17Q-502, as much of the 136Q-502 protein was insoluble and aggregating at the top of the western (Fig. 5*A*). No nonspecific binding of FLAG-ubiquitin was observed with the zero-antibody control (Fig. 5*B*). These 136Q-502 aggregates costained for ubiquitin both in the whole-cell lysate and in the mutant HTT immunoprecipitation westerns, showing that mutant HTT can sequester ubiquitin in aggregates, as observed previously in human brain tissue (26, 49, 50). Our work suggests that the polyQ expansion in mutant HTT may enhance and dysregulate the ubiquitin-binding function of HTT, potentially leading to pathology in HD.

### HTT KO Alters RBP-Containing Stress Granule Clearance and Mitochondrial Health.

Stress granules are membraneless organelles that form upon cellular stress conditions (51) and proteins previously identified to comprise SGs are primarily RBPs (52). Significant enrichment of stress granule-associated RBPs was found within the list of RBPs that comprised the top category among both HTT’s ubiquitinated interactors (Fig. 3 *B* and *C*) and among proteins identified as being degraded by starvation-induced macroautophagy with HTT KO in 8988T cells (Fig. 2*D*). To further evaluate whether HTT plays a necessary role in the trafficking and clearance of RBPs, we evaluated stress granule dynamics in the HTT KO 8988T cells. Stress granule formation was induced by acute sodium arsenite stress. We observed significantly reduced numbers of stress granules with HTT KO in both clonal KO lines compared to control (Fig. 5*C*). This alteration in stress granule clearance dynamics is consistent with the findings that HTT is a critical component of the overall cellular proteostasis machinery, and that loss of function induces proteostatic stress and the up-regulation of macroautophagy, which we observed in these cells (Fig. 1), which may in turn increase stress granule clearance, as with HTT KO we found increased lysosomal RBPs, found in stress granules (Fig. 2*C* and Dataset S2). Finally, the results presented here suggest that HTT may function in appropriate trafficking of ubiquitinated RBPs to the lysosome, with loss of this function producing proteostatic stress and potential impairment of the normal turnover of RBPs if compensatory degradative pathways, like starvation-induced macroautophagy, are not activated, an effect which may be tissue and cell-type specific (31). Alternatively, it is also possible that formation of stress granules is impaired with HTT KO in 8988T cells.

Given that HTT KO showed a reduction of mitochondrial proteins in the lysosomes in our mass spectrometry analysis (Fig. 2*B*) which we suggest may reflect reduced mitophagy, we transfected 8988T parental and clone 10 HTT KO lines with the pMitoTimer plasmid, which encodes a mitochondria-targeted green fluorescent protein when newly synthesized through constitutive expression driven by the CMV promoter. When oxidized, MitoTimer protein shifts irreversibly to red fluorescence (53). We used this reporter to assess mitochondrial health, and observed a significant increase in the red/green ratio with HTT KO (Fig. 5*D*), indicating enhanced mitochondrial oxidative stress and reduced mitochondrial health, consistent with a reduction in mitophagy in 8988T cells with HTT KO.

## Discussion

The goal of this work was to further understand normal HTT function, as a loss of its function with polyQ expansion may contribute to HD pathogenesis (13). Here, we demonstrate that in proliferating cells, HTT copurifies with ubiquitinated or ubiquitin-associated RBPs and that the absence of HTT may result in a potentially compensatory up-regulation of macroautophagy that can then clear these RBPs. This ubiquitin interaction is likely mediated directly by a DisUBM and/or UBM domains within HTT amino acids 235-367, the ubiquitin-binding of which in cells is inhibited by mimicking phosphorylation of HTT S421 and enhanced with polyQ expansion mutation of HTT.

With HTT KO, mitochondrial proteins in purified lysosomes are reduced, mitochondrial oxidative stress is increased, and presumably HTT-mediated basal mitophagy is absent. One of the mitochondrial proteins that fails to target to the lysosome with HTT KO is TFAM, a protein highly enriched in basal autophagosomes in the brain (28). These results are consistent with previous findings that HTT is required for trafficking autophagosomes containing mitochondria (19), and that mitophagy is impaired in HD (5, 54, 55). Our laboratory has recently shown that RNA granules accumulate in mitochondria of HD iPS neurons, which could reflect either reduced HTT function in the clearance of mitochondria due to HTT mutation in HD, or alternatively, changes in mitochondrial protein import (56). 14% of the ubiquitinated and ubiquitin-associated proteins we found copurifying with HTT from St14A cells were mitochondrial, although HTT-mediated mitophagy may not require ubiquitin, as none of these overlapped with mitochondrial proteins that failed to enter the lysosome with HTT KO in 8988T cells. Mitophagy receptor proteins BNIP3 and NIX can interact with the HTT C-terminal autophagic receptor binding domain (5), consistent with a possible role for HTT in ubiquitin-independent mitophagy (57), a function that may not be compensated for by the up-regulation of starvation-induced macroautophagy we observe in the 8988T cells with HTT KO.

The most enriched group of ubiquitinated or ubiquitin-associated proteins that copurified with HTT were RBPs. HTT is not strictly necessary for RBP lysosomal trafficking, in the sense that macroautophagy may compensate by increasing RBPs targeted to the lysosome with HTT KO. However, it is possible that HTT plays a role in selectively trafficking a subset of these ubiquitinated RBPs into the lysosome or endosome under basal conditions. Under stress conditions, HTT may also facilitate the degradation of stress granules through autophagy or the proteasome. 61% of the ubiquitinated/ubiquitin-associated proteins that copurify with HTT are found in the stress granule proteome, 61% in the nucleus, and 50% associated with defective DRiPs (Fig. 3*C* and Dataset S4) (37–39). When mitochondrial and nuclear import is blocked, as can occur with mutant HTT expression (58, 59), DRiPs accumulate in the nucleus in membraneless liquid–liquid phase-separated (LLPS) organelles, and if not cleared properly, form nuclear aggregates, which are a characteristic of HD (38, 50, 60). When we stressed 8988T cells with sodium arsenite, we found significantly reduced numbers of stress granules with HTT KO, consistent with the up-regulated macroautophagic clearance of RBPs we observed in this cell line. Our group previously described an accumulation of G3BP1 granules in the HD patient brain (61). It is possible that in an aged brain with reduced levels of HTT, the compensatory up-regulation of macroautophagy or the proteasome is not available to cope with the inhibitory effect of polyQ expansion on HTT function in RBP autophagic clearance (13, 61). It therefore follows that reduced HTT function from polyQ expansion might result in inadequate autophagic clearance of DRiPs including toxic mutant HTT exon 1 protein (62), particularly with aging, as we find full-length HTT levels decline in the mouse brain with age (13).

We previously found (5, 7) that the C-terminal domain of the HTT protein has similarity in structure and function to the autophagic scaffold Atg11, found to be required for Atg32-dependent mitophagy (63, 64) and for the cytoplasm to vacuole targeting (CVT) pathway, which targets LLPS oligomeric cargo into the yeast lysosome/vacuole in *Saccharomyces cerevisiae* (65). If the CVT pathway becomes impaired, starvation-induced macroautophagy can compensate to target the oligomeric cargo to the yeast vacuole (66). The CVT pathway evolved in *Schizosaccharomyces Pombe* to a form of microautophagy requiring receptor proteins and ubiquitin (67). Here, we propose that ubiquitinated RBPs may be scaffolded by HTT for autophagic degradation by a mechanism similar to the CVT pathway, and if this pathway is blocked by HTT KO, starvation-induced macroautophagy may be up-regulated to compensate, depending on the tissue/cell type. On the other hand, we previously found that like yeast Atg11 interacting with yeast mitophagy receptor Atg32, the C-terminal Atg11-like receptor-binding domain of HTT could bind Bnip3/NIX (5) which function in ubiquitin-independent mitophagy in mammalian cells (57). We propose that KO of HTT may block this pathway which may not be compensated for by up-regulation of starvation-induced macroautophagy in our 8988T cells.

Wt and mutant HTT copurify from the mouse brain with intact ribosomes and inhibit translation (68). More recently, wt HTT has been shown to bind to ribosomes and physiologically inhibit their translocation on mRNA, a function that is further enhanced by mutant HTT leading to slower movement and stalling of ribosomes in HD cells. Depletion of mutant HTT enhances protein synthesis and increases the speed of ribosomal translocation (4). Independently, HTT was shown to interact with the translational complex to regulate translation of specific mRNAs involved in CNS axon regeneration (69). Our data suggests that many RBPs required for translation, including ribosome subunits and elongation factors, may be ubiquitinated and cleared by HTT-mediated selective autophagy. Relevant to HTT’s role in clearing RBPs, mammalian selective autophagy pathways to which HTT may contribute include CMA and endosomal microautophagy. A loss of CMA can be compensated for by an up-regulation of macroautophagy (70), similar to the up-regulation of macroautophagy we observe here with HTT KO. CMA can suppress cellular translation through clearance of RBPs (29), found here to be a major HTT cargo. Lysosomal membrane protein LAMP2A, the CMA receptor protein, is required for clearance of S13 phosphorylated HTT, and of wt and mutant HTT exon 1 protein (7, 14). In addition, CMA is up-regulated in HD models, consistent with the compensatory activation of a mechanism failing in disease (17). Here, we find LAMP2A levels increased with HTT KO in 8988T cells, also consistent with a cellular attempt to up-regulate CMA. CMA is inhibited by the kinase Akt (71), and here we find that the ubiquitin-binding function of HTT is reduced by phosphorylation of HTT S421, an Akt target, as is retrograde vesicular trafficking mediated by HTT (22). We identify that HSC70 (HSPA8), the major chaperone required for CMA and endosomal microautophagy, copurifies with HTT and ubiquitinated proteins. These data further support that HTT may play a role in CMA, a pathway known to clear proteins associated with aggregation in neurodegenerative diseases (72) which may have evolved from the yeast CVT pathway (7). With HTT KO, a compensatory activation of starvation-induced nonselective macroautophagy and proteasome activity may also degrade translation factors and ribosomal subunits to inhibit translation as long as the cell type is able to compensate in this way; however, neurons may be less likely to compensate with an up-regulation of macroautophagy than other cell types (31), which may ultimately contribute to neurodegeneration.

HTT may also play a role in the formation of multivesicular bodies (73–75), or in selective macroautophagy, which both may utilize ubiquitin to identify targets (67, 76). HTT scaffolds retrograde endosomal and autophagosomal transport in neurons (19, 22) toward the cell body, the location of most mature neuronal lysosomes (77). We observed 75% overlap of our ubiquitinated proteins copurifying with HTT 1-502 with the contents of brain-derived autophagosomes (Fig. 3*C* and Dataset S4) (28), consistent with the possibility of HTT participating in selective macroautophagy or autophagosome transport as described (5–7, 19). Dephosphorylation of HTT S421 by calcineurin was recently found to activate HTT’s role in retrograde vesicular transport (22), which is consistent with our finding that phosphorylation of HTT S421 may inhibit its ubiquitin-binding function, while S421 phosphoresistance is associated with enhanced ubiquitin binding in vitro. Thus, HTT’s close proximity with the endosomal surface during this transport could allow it to recruit ubiquitinated proteins to enter the endo/lysosome as it matures on the way to the cell soma.

Phosphorylation of HTT S421 has been shown to be protective against HTT-mediated toxicity and HD progression by many groups (3, 25, 78–82). The ubiquitin-mediated autophagic pathway we describe here, which may be activated by calcineurin-mediated S421 dephosphorylation stimulating retrograde trafficking, might contribute to toxicity in the context of mHTT expression, which enhances interaction with ubiquitinated proteins further and creates a burden for the lysosomal system. In HD, S421 phosphorylated HTT may relieve some strain on the lysosomal system by reducing the activity of this pathway (22). However, this may induce compensatory mechanisms like the proteasome, CMA, or nonselective autophagy to clear S421 phosphorylated HTT and ubiquitinated cargo that does not interact well with S421 phosphomimetic HTT. Recently, neuronal lysosomes containing CMA substrates, including HTT, have been shown to release their contents into the extracellular matrix via lysosome exocytosis where the aggregation-prone cargo may be degraded by the associated lysosomal cathepsins to protect neurons from proteostatic stress (83). As Akt phosphorylation of HTT at S421 can activate anterograde vesicular trafficking mediated by HTT (21) and impact motility of lysosomes (20) it may activate this process resulting in neuroprotection. Similarly, with advancing age, substrates for endosomal microautophagy, a pathway in which HTT may also participate, are secreted when late endosomes release protein-loaded exosomes upon plasma membrane fusion (84), a process that might be enhanced by HTT S421 phosphorylation. Release of S421 phosphorylated HTT-trafficked endosomal/lysosomal contents into the extracellular matrix for degradation may relieve proteostatic stress, potentially consistent with the reduced levels of phosphomimetic HTT fragment we observe in this study. However, this mechanism might ultimately contribute to prion-like propagation of mutant HTT (85).

We find that HTT copurifies with ubiquitinated/ubiquitin-associated RBPs found in stress granules and with VCP, a segregase involved in stress granule clearance by the proteasome (86), and interacts better in vitro with K48 ubiquitin chains preferentially cleared by the proteasome than K63 ubiquitin chains that function in selective autophagy (*SI Appendix*, Fig. S4) (87–89). As HTT interacts with both VCP and p62 (5, 6, 90), proteins involved in both proteasomal and lysosomal degradation of ubiquitinated proteins (91, 92), it is also possible that in addition to being an autophagic scaffold, HTT functions in targeting proteins for proteasomal degradation.

Finally, our study here may direct future research for therapeutic strategies through enhancement of HTT function in the clearance of ubiquitinated cargo, or of compensatory pathways that may clear the accumulating cargo early on in disease, before lysosomal failure occurs with aging and/or with mutant protein expression. We suggest that therapies for HD and other neurodegenerative diseases should consider the potential importance of normal HTT function for proteostatic balance in the brain during aging.

## Materials and Methods

### Reagents.

Plasmids, commercially purchased ubiquitin K48 and K63 chains, and antibodies used in this study are described in **SI Appendix*, *Materials and Methods**.

### Cell Culture and Transfection, Immunoprecipitation, and Western Blot.

Cell culture and lysis of 8988 T cells and St14A cells, coimmunoprecipitation of HTT fragment with ubiquitin, and western blotting techniques are described in **SI Appendix*, *Materials and Methods**.

### Creation of 8988T TMEM192-HA Cells and CRISPR HTT KO in 8988T TMEM192-HA Cells, lysoIP Protocol, and Mass Spectrometry Lysosomal Proteomics in these Cell lines.

The LysoIP tag was engineered into 8988T cells at the *tmem192* locus to create a clonal line of homozygous 8988T TMEM192-HA, and CRISPR was used to generate HTT KO lines in these cells. LysoIP was performed on these cell lines, mass spectrometry proteomic analysis was used, and statistical analysis performed, all described in *SI Appendix*, *Materials and Methods*.

### Cell Biology Analysis.

Lysotracker staining, ubiquitin immunofluorescence, confocal microscopy and image analysis, proteasome activity analysis, stress granule analysis, pMitoTimer analysis, oleic acid treatment, and lipid droplet staining were performed as described in **SI Appendix*, *Materials and Methods**.

### Tandem Purification of HTT Fragment with Ubiquitinated Proteins and Mass Spectrometry for HTT/Ubiquitin Tandem Purification.

HTT 502-HA fragment was copurified with FLAG-ubiquitin using both anti-HA and anti-FLAG immunoprecipitations from St14A cells grown under basal conditions, the copurifying proteins were identified by mass spectrometry, and datasets overlapping with these tandem copurified proteins were determined, as described in **SI Appendix*, *Materials and Methods**.

### M1-Linear Hexa-Ubiquitin Construction and Purification and GST Pull-Down Analysis to Map the Ubiquitin-Binding Domain of HTT.

M1-linear hexa-ubiquitin and HTT GST-fusion proteins were purified from *E. coli* and binding between the two was analyzed as described in *SI Appendix*, *Materials and Methods*

## Supplementary Material

Appendix 01 (PDF)

Dataset S01 (XLSX)

Dataset S02 (XLSX)

Dataset S03 (XLSX)

Dataset S04 (XLSX)

## Data Availability

Mass spectrometry raw data for the Lyso-IP experiments (project accession: PXD053954) (93) and tandem HTT/ubiquitin co-purification (project accession: PXD053980) (94) have been deposited to the ProteomeXchange Consortium via the PRIDE (95) partner repository. All study data are included in the manuscript and/or supporting information.
