# Supplementary material for: Huntingtin contains an ubiquitin-binding domain and regulates lysosomal targeting of mitochondrial and RNA-binding proteins
Source: Proc Natl Acad Sci U S A. 2024 Jul 29;121(32):e2319091121. doi: 10.1073/pnas.2319091121 (PMC11317567; doi:10.1073/pnas.2319091121)
Supplement: Supplementary file 1 — Appendix 01 (PDF) [file pnas.2319091121.sapp.pdf]

PNAS

Supporting Information for

Huntingtin contains an ubiquitin-binding domain and regulates lysosomal targeting of mitochondrial and RNA-binding proteins

Gianna M. Fote, Vinay V. Eapen, Ryan G. Lim, Clinton Yu, Lisa Salazar, Nicolette R. McClure, Jharrayne McKnight, Thai B. Nguyen, Marie C. Heath, Alice L. Lau, Mark A. Villamil, Ricardo Miramontes, Ian H. Kratter, Steven Finkbeiner, Jack C. Reidling, Joao A. Paulo, Peter Kaiser, Lan Huang, David E. Housman*, Leslie M. Thompson, Joan S. Steffan*

*Co-Corresponding authors:

David E. Housman, Ph.D.
Email: dhousman@mit.edu

Joan S. Steffan, Ph.D.
Email: jssteffa@uci.edu

This PDF file includes:

SI Materials and Methods
Figures S1 to S10
Legends for Datasets S1 to S4
SI References

SI Materials and Methods:

Creation of PATU8988T TMEM192-HA cells

To engineer the Lyso-IP tag into 8988T cells using homology-directed repair, a gene block encoding a 3xHA epitope tag, a puromycin cassette, and homology arms on either side of the cleavage site was synthesized by Integrated DNA Technologies to edit the *tmem192* locus, as previously described (1). This sequence was cloned into the pSmart (Lucigen Cat#40041–2) shuttle vector using Gibson assembly (New England Biolabs). The shuttle vector along with the TMEM192 sgRNA sequence along with the shuttle vector was transiently transfected into 8988T cells and puromycin selection was performed 5 days post-transfection for 7–8 days. The mixed pool of cells that were puromycin resistant were single-cell plated and clonal lines of homozygous 8988T TMEM192-HA were isolated.

CRISPR HTT knockout in PATU8988T TMEM192-HA cells

HTT knockout lines were generated using an optimized strategy previously reported by Ruzo et al. (2) that combined two gRNAs flanking HTT exon1: CTGCTGCTGGAAGGACTTGA and GCTGCACCGACCGTGAGTTT. CRISPR-Cas9 ribonucleoprotein (RNP) complexes containing both gRNAs and high-fidelity (HiFi) Cas9 protein (IDT) at a molar ratio of 1.2:1.0 gRNA:Cas9 were delivered to 8988T cells by reverse transfection using Lipofectamine RNAiMAX transfection reagent (Invitrogen). Transfected cells were allowed to recover overnight, then dissociated for single-cell plating in 96-well plates. Clonal colonies were visually identified for expansion and PCR-based screening using primers AGAGCCCCATTCATTGCCCC (Forward) and GGTTGCTGGGTCACTCTGTCT (Reverse), spanning the targeted region. PCR products positive for editing were further evaluated by Sanger sequencing and Western to confirm knockout of HTT protein. Three clones (KO10, KO59, and KO73) were chosen for use.

Cell culture and transient transfection

All cell lines were maintained in DMEM (Corning 10-017-CV) supplemented with 10% FBS (ThermoFisher 26140079). PATU8988T (8988T) cells stably expressing TMEM192-HA were maintained in DMEM (Corning 10-017-CV) supplemented with 10% FBS (ThermoFisher

26140079) and cultured at 37 degrees C, 5% CO_2. ST14A cells (3) were cultured at 33 degrees C and 5% CO_2. For transient transfection of ST14A, cells were forward transfected using Lipofectamine 2000 (ThermoFisher 11668027) 24 hours later as instructed by manufacturer. Transfection reagents were incubated in the bottom of a 6 well plate and cells were plated directly on transfection mix. Media was changed 24 hours after transfection, and cells were harvested for western blot 48 hours after transfection. Bafilomycin A1 (Cayman Chemical 11038) was used at 50nM for 4 hours before harvesting cells for western blot. Cells were treated with HBSS for four hours before harvest to induce starvation response.

LysoIP Protocol

8988T TMEM192-HA cells were plated 750,000 cells per plate onto 150mm dishes (Fisher Scientific 08-772-24) with 2 (WT) or 3 (HTT KO lines) replicate plates per conditioned media group. Twenty four hours later, cells were treated with 50 nmol Baf for 4 hours and lysed using a dounce homogenizer. LysoIP was performed as previously described (4).

Mass spectrometry lysosomal proteomics in PATU8988T cells

Protein extracts were subjected to reductive alkylation with 5mM DTT incubated at 55°C for 30 mins while shaking at 900 rpm. The protein mixture was allowed to cool to room temperature, followed by incubation with 15mM iodoacetamide, 50mM ammonium bicarbonate solution at room temperature in the dark for 30 minutes while shaking at 900rpm. The reaction was quenched by addition of 10mM DTT, incubated at room temperature in the dark for 15 minutes. TCA precipitation was then performed, with 1 part 100% TCA added to 3 parts sample (final TCA concentration 25%). Samples were vortexed and incubated on ice overnight. Samples were then spun at maximum speed on a tabletop centrifuge at 4°C for 30 minutes and washed three times with ice cold methanol. Samples were dried in a speedvac, resuspended in 200 mM EPPS pH8.0, and digested at room temperature overnight shaking with LysC protease, 0.005U/ µL. Trypsin was then added at 500ng/µL and the reaction was incubated for 6 h at 37°C. Following digestion, acetonitrile (ACN) was added (30 µL per 100 µL digestion) and incubated for 10 minutes at room temperature. Tandem mass tag (TMT) solutions in anhydrous ACN were warmed at room temperature for 15 minutes, spun, and 4 µL added to each sample and incubated for 1 hour

at room temperature. The reaction was quenched with hydroxylamine with a final concentration of 0.5% (v/v) for 15 min at room temperature. The TMT-labeled samples were then pooled at a 1:1 ratio. The sample was vacuum centrifuged in a speedvac. Dried TMT-labeled sample was resuspended in 100 µL of 5% ACN, 5% formic acid solution. pH was adjusted to 2 using formic acid. Samples were fractionated according to manufacturer's instructions using High pH reversed-phase peptide fractionation kit (Thermo Fisher Scientific). Over a 90 min run, samples were offline fractionated and then pooled. Fractions were dried in a speedvac. Each fraction was desalted using StageTip, dried, and reconstituted in 5% acetonitrile, 5% formic acid solution for LC-MS/MS processing. An Orbitrap Fusion Lumos mass spectrometer (Thermo Fisher Scientific, San Jose, CA) coupled to a Proxeon EASY-nLC1200 liquid chromatography (LC) pump (Thermo Fisher Scientific) was used to collect mass spectrometry data, as described previously (5). Mass spectra were processed using in-house software pipeline as described previously (5). Database searches included all entries from the Human Reference Proteome (2017-05) UniProt database and an in-house curated list of contaminants, concatenated with a database of all protein sequences in reverse order. Searches were performed using a 20 ppm precursor ion tolerance, and the product ion tolerance was set to 0.9 Da. Peptidespectrum matches were adjusted to 1% false discovery rate. Peptide spectrum match filtering was performed using a linear discriminant analysis, as previously described (6) and the following parameters were considered: DCn (or Diff Seq. Delta Log Expect), XCorr (or Comet Log Expect), peptide length, charge state, missed cleavages, and precursor mass accuracy. We extracted the summed signal-to-noise ratio for each TMT channel, and found the centroid that most closely matched the expected mass of the TMT reporter ion, with integration tolerance of 0.003 Da. Protein spectra matches with poor quality, TMT reporter summed signal-to-noise ratio less than 150, MS3 spectra with isolation specificity less than 0.7 or no MS3 spectra were excluded from quantification. For differential protein abundance, this mass spectrometry data was analyzed using Bioconductor libraries (e.g. limma and edgeR) and methods similar to those reported in many prior publications (7-10).

Lysotracker staining

8988T cells were incubated with Lysotracker Red DND-99 (ThermoFisher Scientific L7528) 75 nM diluted in media for 30 minutes. Cells were then fixed, washed with PBS, and co-stained with DAPI.

Ubiquitin immunofluorescence

Cells were fixed in 4% PFA (Fisher Scientific 50980487) for 15 minutes and permeabilized in 0.3% triton-X-100 for 10 minutes at room temperature. Cells were then blocked in 10% normal goat serum, 0.1% Triton-X-100. Cells were incubated in primary antibody (Invitrogen 13-1600) overnight at 4 degrees C. Cells were then washed three times in PBS and incubated in Alexa-fluor 488 anti-mouse secondary antibody for 1 hour in the dark at room temperature. Cells were again washed 3 times, stained with DAPI for 10 minutes, and imaged using confocal microscopy.

Confocal Microscopy and Image analysis

Cells were fixed in 4% PFA (Fisher Scientific 50980487) for 15 minutes. Fixed cells were imaged on an Olympus FV3000 microscope at 20x. 3 images were collected from at least 3 wells per treatment or genotype. Imaris analysis software was used to quantify number and size of bodipy and lysotracker spots. DAPI spots were used to normalize to cell number for each image.

Proteasome activity assay

Fluorescence-based 20S proteasome activity assay was performed as instructed by the manufacturer (Cayman Chemical 10008041). Six replicate wells were analyzed for each cell line-parental and three HTT KO cell lines. Cells were treated with Baf or no treatment for 4 hours at 50 nM. Fluorescence was analyzed using a Biotek plate reader.

Oleic Acid treatment and Lipid droplet staining

8988T cells were treated with 200 μM oleic acid (Millipore Sigma CAS 112-80-1) conjugated to BSA as previously described (11) overnight to stimulate lipid droplet formation (12). Lipid droplets were stained using a 2 μM solution of Bodipy 493/503 (Thermo Fisher D3922) as previously described (13).

Western blot

Cells were harvested using lysis buffer containing 10% glycerol, 20mM Tris pH 7.5, 137mM NaCl, 1% NP40, 5mM EDTA, phosphatase inhibitors 2 (Millipore Sigma, P5726) (1:1000) and 3 (Millipore Sigma P0044) (1:1000), 5mM nicotinamide (Sigma N3376), 5 mM butyric acid, 1mM PMSF, 10 µg/mL aprotinin, 10 µg/mL leupeptin, and one Pierce mini protease pellet (Fisher Scientific A32953) per 10 mL of lysis buffer. Lysates were sonicated and 20µg of protein was then used for SDS/PAGE. NuPage Novex 4-12% Bis-Tris precast gel (Life Technologies WG1402BOX) was used with MOPS running buffer (Invitrogen NP0001); NuPage 3-8% Tris-Acetate Midi protein gels (Thermo Fisher Scientific WG1602BOX) were used with Tris-Acetate running buffer (Fisher Scientific LA0041). Protein was then transferred onto Immobilon-FL PVDF (Millipore Sigma IPFL00010) or 0.2µ nitrocellulose (BioRad). Whole protein was quantified using a revert assay (LI-COR Biosciences 926-11016), and the membrane was blocked with Intercept (TBS) Blocking Buffer (LI-COR biosciences 927-60010) for 1 hour. The membrane was then incubated in primary antibody overnight, washed three times with TBS-0.1% Tween-20, and incubated for 1 hour in near-infrared-conjugated secondary antibody in intercept block supplemented with 0.1% Tween-20. Membranes were imaged on a LI-COR scanner and quantified using Empiria Software.

Statistics for LysoIP and HTT KO cell experiments

Each reporter ion channel was summed across all quantified proteins and normalized with the assumption that protein loading was equal across all samples. The unit of protein measurement analyzed statistically was the signal to noise value of the TMT label for a particular peptide.

Data were log transformed and examined for normality using histogram plots and testing with Shapiro-Wilk's method. Both revealed the data to fit a normal distribution allowing for use of downstream linear models. Next, differential statistics using a mixed linear model on library size normalized and log transformed values were used. For this analysis two HTT knockout cell lines were combined as one group since these represent biological clones.

Furthermore, the statistical design included batch as a random effect and treatment as a fixed effect. Using this model differential abundance between HTT and wild type samples were examined using untreated and treated samples, the treatment effects within group, and the interaction between genotype and treatment. The Benjamini-Hochberg method was used to correct for multiple tests and statistical significance was determined using either a p-value < 0.05 or a false discovery rate < 10%. For biological interpretation EnrichR analysis was performed on proteins that were significantly increased or significantly reduced separately, to determine significantly altered pathways and GO terms among proteins enriched or depleted in the lysosome. For Ingenuity Pathway Analysis (IPA), all significantly altered proteins were analyzed to identify significantly altered pathways. Statistics were performed using PRISM software. All experiments were performed with at least 3 biological triplicates (3 separate cell culture wells). Although mass spectrometry experiments were constrained by number of samples that could be analyzed in each batch and therefore we were only able to use two HTT KO clones, we were able to use three HTT KO clones in subsequent experiments. Since these clones are theoretically identical biological replicates, samples from each clone line in triplicate were combined for statistical analysis, resulting in n of three times greater sample number for HTT KO than for parental. Error bars represent standard error of the mean. One-way ANOVA with multiple comparisons was corrected using Tukey's correction. For western blot and qPCR experiments Student's Two-Tailed T test was used. For Incucyte analysis, two-way ANOVA with mixed effect model repeated measures was corrected for multiple comparisons by controlling the False Discovery Rate with the two-stage step-up method of Benjamini, Krieger, and Yekutieli. Significant P values are indicated as follows: $p<0.05$ *, $p<0.01$ **, $p<0.001$ ***, $p<0.0001$ ****.

Tandem Purification of HTT fragment with ubiquitinated proteins

Purifications were done 3 independent times. St14A cells were transiently co-transfected with 17Q-HTT 502 aa-HIS-HA-HA-HIS (H4 tag) or vector pcDNA3.1 control, together with FLAG-ubiquitin encoding plasmid. For transfection, total DNA is 1 µg, 0.5 µg for 1-502 HTT-HIS-HA-HA-HIS plasmid, and 0.5 µg for FLAG ubiquitin plasmid. Cell density is 1 x 10 5 cells per well in a 6 well plate, with growth area ~9.6 cm2. Two days after transfection, *in vivo*

formaldehyde (0.05%) cross-linking of intact cells was carried out in PBS buffer at room temperature for 10 minutes and quenched with a final concentration of 0.125 M glycine as previously described (14). The cross-linked cells were washed with PBS then broken in break buffer [20 mM Tris·HCl, pH 7.5, 10% (vol/vol) glycerol, 137 mM NaCl, 10% glycerol, 1% Nonidet P-40, supplemented with 20 mM Nethylmaleimide, 1 mM PMSF, phosphatase inhibitors 2 and 3 (Sigma-Aldrich), 10 ng/mL aprotinin, 10 ng/mL leupeptin and EDTA-free liquid protease inhibitor (EMP Millipore 539134)]. Cell lysates were sonicated 3 x 10 seconds on ice, then microfuged at 4 degrees C 20 minutes at rcf 20,000. Two independent purifications (as in Figure 3A): The supernatant was incubated with ANTI-FLAG M2 Affinity Gel beads (Sigma A2220) that had been pre-equilibrated in break buffer, and rotated overnight at 4 degrees C. The following day, the beads were microfuged at 5200 rpm for 30 seconds, then washed 4 times (5 minutes rotating at 4 degrees C between microfugations) in break buffer without glycerol (wash buffer). Immunoprecipitated FLAG-ubiquitin conjugates were eluted from the beads with 0.15mg/ml FLAG peptide (Sigma F3290) 2 x 30 minutes 4 degrees C rotating. The FLAG eluate was then incubated overnight at 4 degrees C rotating with Anti-HA Affinity Matrix beads (Roche, 11 815 015 001) pre-equilibrated in wash buffer. The beads were washed the following day 4 x 5 minutes rotating at 4 degrees C with microcentrifugation 2 minutes x 2000 rpm. Purified proteins were eluted from the beads with 0.1M glycine pH 2 rotated 20 minutes at room temperature. The eluate was frozen and sent for mass spectrometry analysis. Third independent purification: The supernatant was incubated with Anti-HA Affinity Matrix beads (Roche, 11 815 015 001) that had been pre-equilibrated in break buffer, and rotated overnight at 4 degrees C. The following day, the beads were microfuged at 2 minutes x 2000rpm, then washed 4 times (5 minutes rotating at 4 degrees C between microfugations) in break buffer without glycerol (wash buffer). Immunoprecipitated HTT-HIS-HA-HA-HIS conjugates were eluted from the beads with 1mg/ml HA peptide (Roche 11666975001) 2 x 30min 4degrees C rotating. The HA eluate was then incubated overnight at 4 degrees C rotating with ANTI-FLAG M2 Affinity Gel beads (Sigma A2220) pre-equililbrated in wash buffer. The beads were washed the following day 4 x 5 minutes rotating at 4 degrees C with microcentrifugation 5200 rpm for 30 seconds. Purified proteins were eluted from the beads with 0.1M glycine pH 2 rotated 20 minutes at room temperature.

Mass Spectrometry for HTT/ubiquitin tandem purification

Tandem-purified proteins were digested via filter-aided sample preparation (FASP). Microcon-10kDa centrifugal filters were first used to concentrate eluants, followed by cysteine reduction and alkylation using 5 mM tris (2-carboxyethyl) phosphine (TCEP) at RT for 30 minutes and 10 mM iodoacetamide at RT in the dark for 30 minutes, respectively. Proteins were then digested on-filter in 50 mM ammonium bicarbonate pH 7.8 with 1.5M urea using trypsin overnight at 37 degrees C. The resulting peptides were then cleaned using Sep-Pak C18 cartridges prior to LC MS/MS analysis using an UltiMate 3000 RSLC (Thermo Fisher Scientific) coupled on-line to an Orbitrap Fusion Lumos™ mass spectrometer (Thermo Fisher Scientific). Reverse-phase separation was performed on a 50 cm x 75 µm I.D. Acclaim® PepMap RSLC column. Peptides were eluted using a gradient of 4% to 25% B over 87 minutes at a flow rate of 300 nl/minutes (solvent A: 100% H_2O, 0.1% formic acid; solvent B: 100% acetonitrile, 0.1% formic acid). Each cycle consisted of one full Fourier transform scan mass spectrum (375–1800 m/z, resolution of 60,000 at m/z 400) followed by 10 data-dependent MS/MS acquired in the linear ion trap with higher collision-induced dissociation (HCD) using NCE (normalized collision energy) 30%. Target ions already selected for MS/MS were dynamically excluded for 30s.

Protein quantitation of LC MS/MS data was carried out using MaxQuant as previously described (15). Briefly, raw spectrometric files were searched using MaxQuant (v. 1.6.0.16) against a complete human proteome obtained from SwissProt (20,520 entries; version from July, 2020). MS/MS spectra were filtered to contain at most eight peaks per 100 mass unit intervals. The first search peptide tolerance was set to 20 ppm, with main search peptide tolerance set to 4.5 ppm. Both peptide spectrum match and protein FDRs were set at 1%, in razor peptide fashion. Trypsin was selected for the protease with up to 2 missed cleavages; no nonspecific cleavage was allowed. For protein quantitation, cysteine carbamidomethylation was set as a fixed modification, while methionine oxidation and N-terminal acetylation were selected for variable modifications, maximum of 2 per peptide. Intensities were determined as the full peak volume over the retention time profile. Intensities of different isotopic peaks in an isotope pattern were always summed for further

analysis. "Unique plus razor peptides" was selected as the degree of uniqueness required for peptides to be included in quantification.

Count level protein quantification values were then used for further exploration and statistical analyses. Count data were log2 transformed and normalized to library size. Proteins were removed if not present in at least 2 of the total samples analyzed. After confirmation that the protein count distributions fit a gaussian curve these data were then used for differential statistics. Differential analysis was performed on the remaining proteins using the Bioconductor package Limma (https://doi.org/10.1093/nar/gkv007). Statistically significant differential proteins were determined using an adjusted p-value (False discovery rate) of <0.1. This set of proteins were used for additional GO and pathways analyses using EnrichR. Significant GO terms and pathways were identified using a FDR of <0.1. Additionally, String protein-protein interaction analysis was conducted on the 205 Ub Co-purification set of proteins. To identify the PPI networks between these proteins we set the parameters for analysis to only include physical interactions validated from experimental analysis or databases, with a high confidence score >0.7.

Analysis of overlapping protein datasets

The MIT Bioinformatics Compare website (http://barc.wi.mit.edu/tools/compare/) was used to compare lists of published proteins with co-IP dataset to identify proteins found in both data sets. Percentage of co-IP proteins found in published datasets are presented.

Immunoprecipitation

5 x 6 well plates of St14A cells were co-transfected with 17Q-502-HIS-HA-HA-HIS and FLAG-ubiquitin plasmids and were lysed (2 wells/sample) after two days in 20 mM Tris·HCl, pH 7.5, 10% (vol/vol) glycerol, 137 mM NaCl, 1% Nonidet P-40, supplemented with 1 μg/ml leupeptin and aprotinin, 20 mM N-ethylmaleimide, 1 mM PMSF, phosphatase inhibitors 2 and 3 (Sigma-Aldrich), and EDTA-free protease inhibitors (Thermo Scientific A32965). Lysates were sonicated 3 x 10 seconds on ice, then protein was quantitated by Bradford analysis. Equal volumes (~300 μg each) of lysate was immunoprecipitated with 1 μl of either

anti-HA MAB (Covenance) or anti-FLAG MAB (Invitrogen) in 500 µl total volume with 30 µl of Protein G Dynabeads (1004D, Invitrogen) and incubated rotating at 4 degrees Celsius overnight. Samples were washed 3 x in 500 µl the same buffer without glycerol, and run on 4-12% MOPS gels (Thermo Fisher) as described in the Western Blot protocol above. Gels were blotted to either Immobilon-FL (Millipore Sigma IPFL00010) or .2 µm nitrocellulose membrane (BioRad), stained with revert, destained, blocked in LI-COR Intercept (TBS) blocking buffer, and then incubated overnight with 1:500 anti-HA rabbit antibody (Sigma) and 1:1000 anti-FLAG (Invitrogen) antibody, and detected with LI-COR secondary antibodies. Data was analyzed using Empirica Studio (LI-COR). To calculate relative ubiquitin co-immunoprecipitation of HTT, levels of HTT were detected with rabbit polyclonal anti-HA antibody (Sigma) and a ratio of these levels was made for anti-FLAG/anti-HA to compare levels of HTT coming down with FLAG-ubiquitin vs. levels of HTT coming down itself with anti-HA antibody from the same lysate volume. Experiments were done in triplicate and repeated at least twice. To calculate relative levels of FLAG-ubiquitin pulled down with immunoprecipitated 17Q vs. 136Q 502-HIS-HA-HA-HIS, levels of FLAG-ubiquitin detected in anti-HA IP were compared with levels of FLAG-ubiquitin detected in whole cell lysate using anti-FLAG MAB (Invitrogen).

Plasmid construct generation

Generation of p62 UBD construct: The Takara Bio InFusion cloning system was used to clone the p62 UBD (16) from a myc-p62 construct into a pGex2T vector with a GST tag for bacterial expression. Inverse PCR was used to linearize the vector. Gene-specific primers with 15 bp extensions homologous to the ends of the linearized vector were designed, and used to amplify the p62 UBD. The InFusion cloning kit protocol was used as follows. Cloning enhancer was added to PCR product and it was incubated for 15 minutes at 37 degrees C and 15 minutes at 80 degrees C. Reagents for the InFusion cloning reaction were then combined, including the linearized vector, the PCR amplified insert, Enzyme Premix, and water, and the cloning reaction was incubated for 15 minutes at 50 degrees C. Fifty µl of Stellar competent bacteria were thawed on ice and transformed with 2.5 µl of cloning mixture in a round-bottomed falcon tube. Tubes were incubated on ice for 30 minutes, heat shocked for 45

seconds at 42°C, rested on ice for 2 minutes, then 500μL of pre-warmed SOC medium added. This was incubated in a shaker at 37 degrees C for 1 hour, and then 200 μl plated on antibiotic resistance LB plate. *Generation of GST-HTT fusion proteins and HTT constructs used for cellular expression:* GenScript was commissioned to create GST-235-502aa HTT wt and mutant constructs in pGEX-2TK and HTT 17Q- and 136Q-502 and 586 wt and mutant constructs C-terminally tagged with HIS-HA-HA-HIS in pcDNA3.1 for cellular expression. For Figure S3, constructs were created in house. All sequences were verified by Eurofins Genomics sequencing.

M1-linear hexa-ubiquitin construction and purification

The head to toe hexa-ubiquitin construct was generated by subcloning the human ubiquitin cDNA into a modified pET28(a) vector with an N-terminal 6xHis-MBP and C-terminal 6xHis. Engineered SalI and XhoI restriction sites were used, and glycine 76 was mutated to a valine by PCR. A linker region with a sequence of VLDGGGGSGGGGS was inserted between each ubiquitin. Sequencing was used to confirm insertion. Ubiquitin I44A and S65E were constructed in the same manner. For protein production, Rosetta (DE3) cells were transformed with the ubiquitin constructs. The cells were grown in LB media at 37 degrees C with antibiotics kanamycin and chloramphenicol to an OD600nm ~0.4, the temperature was decreased to 16 degrees C and the expression was induced with 0.2 mM IPTG overnight. The cells were harvested and resuspended in lysis buffer containing 50 mM Tris, 250 mM NaCl, 2 mM PMSF, 10 mM imidazole, and 0.1 % Triton X-100, pH 8.0. The cells were sonicated on ice and the lysate was clarified by centrifugation at 4 degrees C for 20 minutes. The supernatant was incubated with Ni sepharose (Cytiva) for 1 hour at 4 degrees C with constant tumbling before loading the Ni sepharose on a gravity column. The column was washed with 50 mM Tris (pH 8.0), 250 mM NaCl, and 20 mM imidazole. The recombinant proteins were eluted with 50 mM Tris (pH 8.0), 250 mM NaCl, and 250 mM imidazole. Protein samples were separated on a 10% SDS-PAGE and stained with Coomassie to determine purity. Pure fractions were collected and combined. Hexa-ubiquitin constructs were used for binding to increase avidity.

GST pull-down analysis

Linear hexa-ubiquitin tagged with His was produced in Rosetta cells (Novagen), purified using nickel column, and eluted using imidazole. GST-proteins were produced in Rosetta cells using IPTG (Millipore Sigma) induction, purified, and bound to glutathione-sepharose 4B beads (Sigma-Aldrich GE17-0756-01). Beads were incubated in buffer containing 20mM Hepes-KOH pH 7.6, 100mM NaCl, 0.1% TritonX-100, 10% glycerol, PMSF and 0.2% BSA. 30μL of 50% bead slurry was diluted in 200 μL of buffer containing purified ubiquitin for 1 hour, then beads were washed 3 times in buffer without BSA or ubiquitin, denaturing loading buffer was added to beads and protein, samples were boiled for 5 minutes and analyzed by western blot using hand-poured 12% gels. Protein was then transferred onto 0.2μ nitrocellulose (BioRad). Whole protein was quantified using revert assay (LI-COR Biosciences 926-11016), and the membrane was blocked with Intercept (TBS) Blocking Buffer (LI-COR biosciences 927-60010) for 1 hour. The membrane was then incubated in primary mouse monoclonal anti-ubiquitin antibody overnight, washed three times with TBS-0.1% Tween-20, and incubated for 1 hour in near-infrared-conjugated secondary antibody in intercept block supplemented with 0.1% Tween-20. Membranes were imaged on a LI-COR scanner and quantified using Empiria Software. In figure S3, GST-bound proteins were first quantified using a gradient of protein run on a hand-poured gel and stained with Coomassie blue. Binding assay was performed as above except that incubation of ubiquitin with GST-linked ubiquitin binding proteins was performed at room temperature for 30 minutes. PVDF membrane was used for protein transfer and blocked with StartingBlock buffer (Thermo Fisher37538). Western blot was developed using chemiluminescent Pico and Dura substrate (Thermo Fisher 34579 and 34075) on film in a dark-room.

Stress granule analysis

8988T cells with and without HTT KO were plated, and at 80% confluency were treated with 125 μM Sodium Arsenite (Sigma Aldrich) for 90 minutes to induce an oxidative stress phenotype. Cells were stained and processed for fluorescence microscopy as previously described (17) utilizing a 1:1000 dilution of the G3BP1 antibody (MBL, RN048PW). Quantitation of cells with G3BP1-positive stress granules (SGs) and Hoechst markers were

captured. SG and nuclei quantification were completed using Cell Profiler. A parameter was created which normalized the SG count to nuclei in a single image to help control for differences in cellular confluency attributed to stressors.

MitoTimer analysis

8988T cells (Creative Bioarray cat.no. CSC-C0307), parental and HTT knock out lines, were maintained in DMEM + 5% HI FBS + 5% HI HS. Cells were plated in a 12 well plate (3 wells/line), at 1 x 10^5 cells/well, on a Poly-D-lysine coated coverslip in each well, and cultured overnight. Cells were transfected with 1 μg of pMitoTimer (Addgene cat.no. 52659) (18) and 2 μl of Lipofectamine 2000 (ThermoFisher cat.no. 13778150) per well for 16 hours. Coverslips were fixed in 4% PFA in PBS for 15 mines on ice, and were washed with cold PBS. Coverslips were imaged by a Zeiss LSM 900 microscope with Airyscan using a 63x objective. Images are analyzed by Imaris software 10.1.1, using the surface module to measure the sum of green intensity and the sum of red intensity of each cell (18).

Statistics

All cell culture and GST pull-down experiments were performed at least twice in triplicate. Statistics were performed using PRISM software. Error bars represent standard error of the mean. For multiple comparisons One-Way ANOVA was employed. Pairwise statistical analysis was achieved by unpaired t-test. Significant p-values are indicated as follows: $p<0.05$ *, $p<0.01$ **, $p<0.001$ ***, $p<0.0001$ ****.

Antibodies

Target	Company/ Catalogue #	Concentration
HA, rabbit polyclonal	Sigma Aldrich H6908	1:500
HA, MAB	Biolegend 901502	1:1000
Ubiquitin, MAB	Invitrogen 13-1600	1:500
FLAG, MAB	Invitrogen F3165	1:1000
LAMP2A,rabbit monoclonal	Boster M01573	1:500
P62/SQSTM1	Abnova PAB16850	1:1000
HTT, rabbit polyclonal	Abcam EPR5526	1:1000
G3BP1, rabbit polyclonal	MBL RN048PW	1:1000
Secondary mouse HRP	Jackson 115035146	1:20,000
Secondary Goat anti-rabbit 800	LI-COR 926-32211	1:20,000
Secondary Goat anti-rabbit 700	LI-COR 926-68071	1:20,000
Secondary Goat anti-mouse 800	LI-COR 926-32210	1:20,000
Secondary Goat anti-mouse 700	LI-COR 926-68070	1:20,000
Secondary for ubiquitin IF	Alexa-fluor 488 anti-mouse	1:1000
Primary for ubiquitin IF	Invitrogen 13-1600	1:250

Purified Ubiquitin

Boston Biochem Tetra-Ubiquitin/Ub4 WT Chains (K48-linked) catalogue UC-210B

Boston Biochem Tetra-Ubiquitin/Ub4 WT Chains (K63-linked) catalogue UCN-310

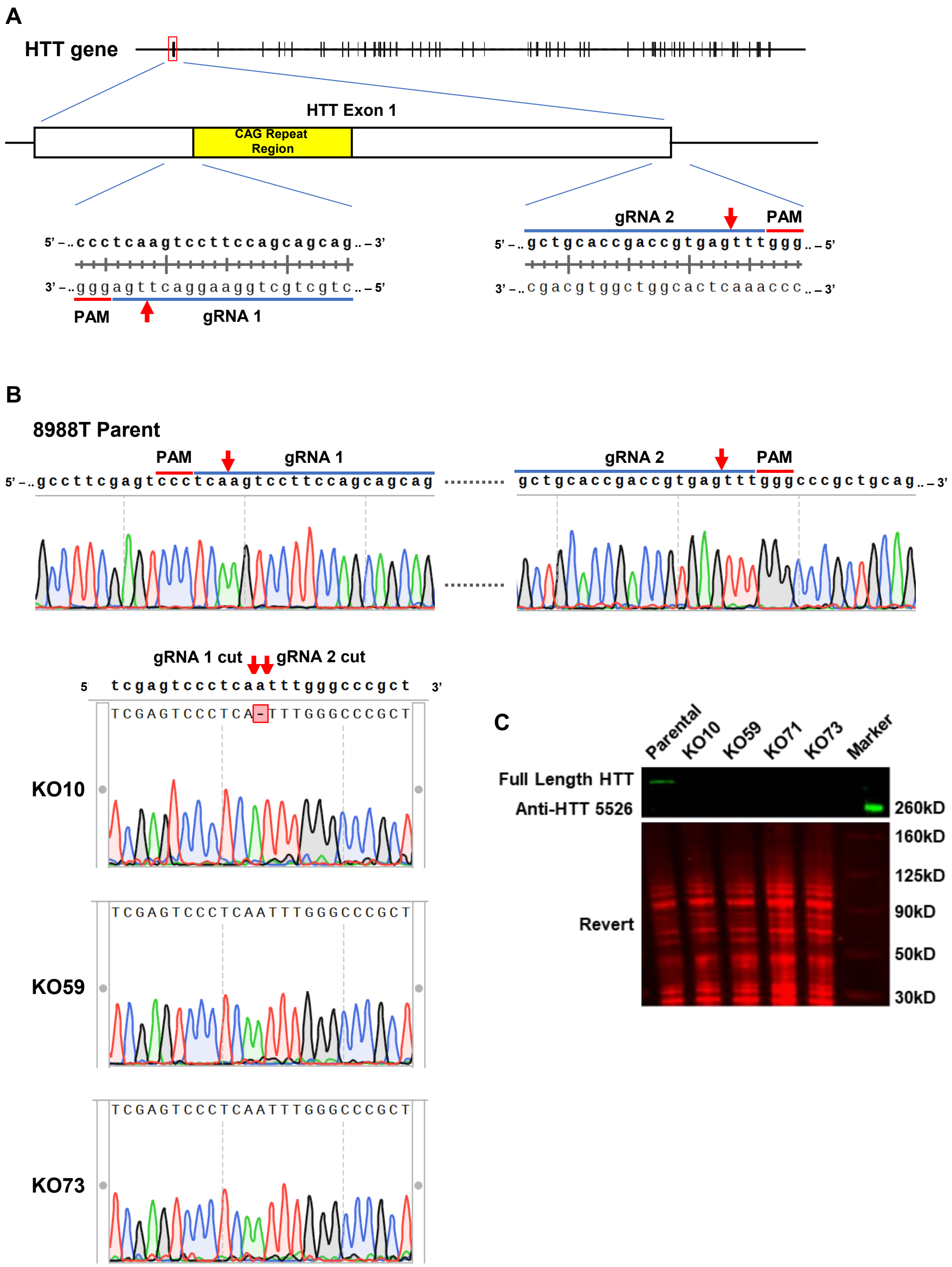

Fig. S1. Generation and validation of 8988T HTT knockout lines. (A) Strategy for generating HTT KO 8988T lines using a pair of gRNAs to specifically delete HTT exon1. gRNA sequences are underlined in blue, and PAM sequence in red. The predicted Cas9 cleavage site is indicated by the red arrow. (B) Sanger sequencing of parental and KO clones. Both alleles appear to have the same edit, resulting in a 218bp (KO59, KO73) or 219bp (KO10) deletion. (C) Western analysis with anti-HTT 5526 antibody of parental cell line and 4 HTT KO lines (KO 10, KO59, KO71, KO73) demonstrates no HTT immunoreactivity in HTT KO lines.

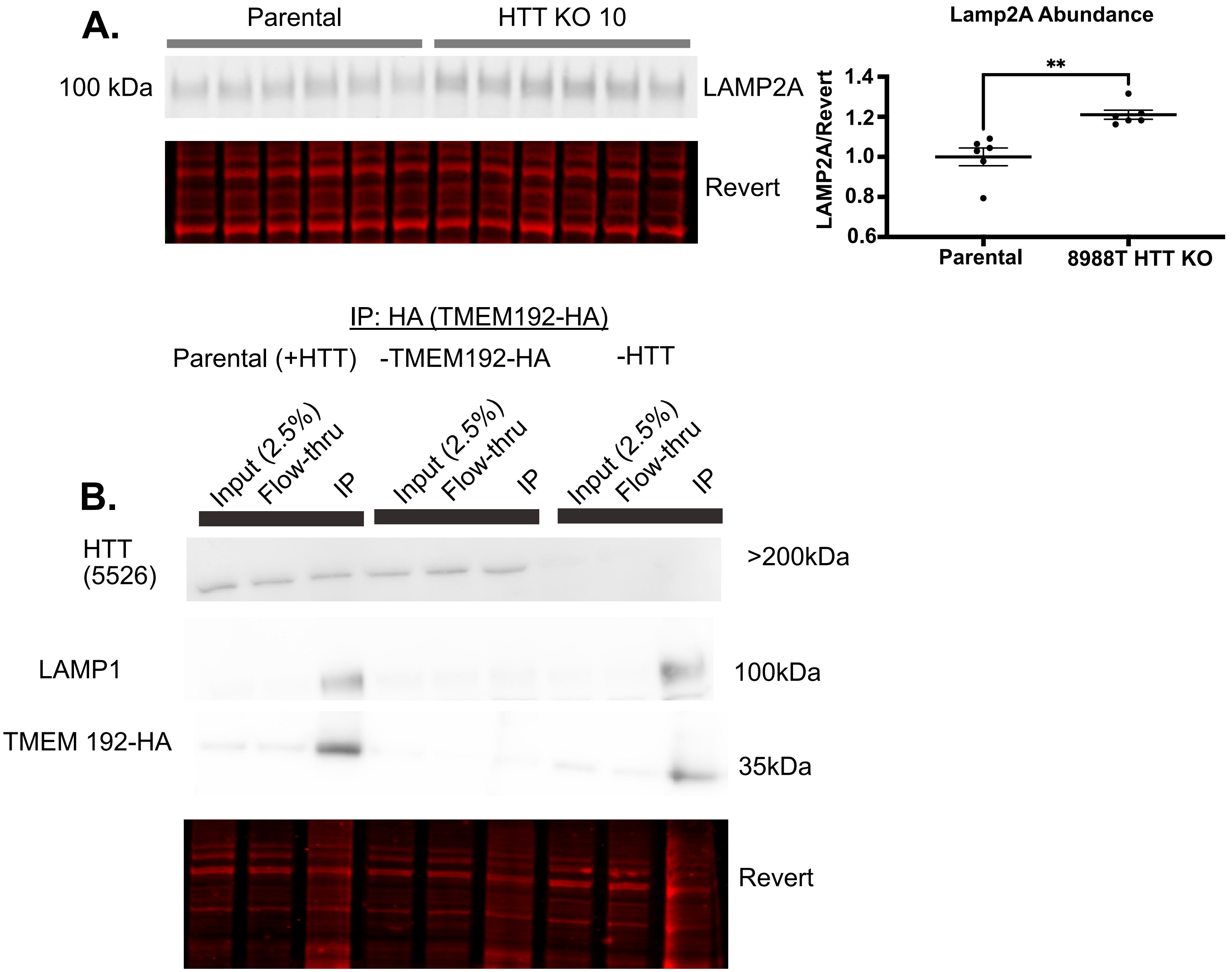

Fig. S2. Analysis of lysosomes in HTT KO cells. (A) In HTT KO 8988T cells, LAMP2A levels are significantly reduced by unpaired t-test compared with parental control. (B) Validation of Lyso-IP in 8988T cells. LysoIP was performed in 8988T cells expressing TMEM192-HA using magnetic HA pulldown beads. Western blot was performed on input (whole cell), flow-through, and lysosomal eluate of three cell lines, the parental cell line expressing HTT and TMEM192-HA, the negative control cell line not expressing the lysosomal tag, and one of the two HTT KO cell lines with no immunoreactive HTT signal on the western. Lamp1 antibody was used as a lysosomal marker. HTT knockout was verified using HTT antibody.

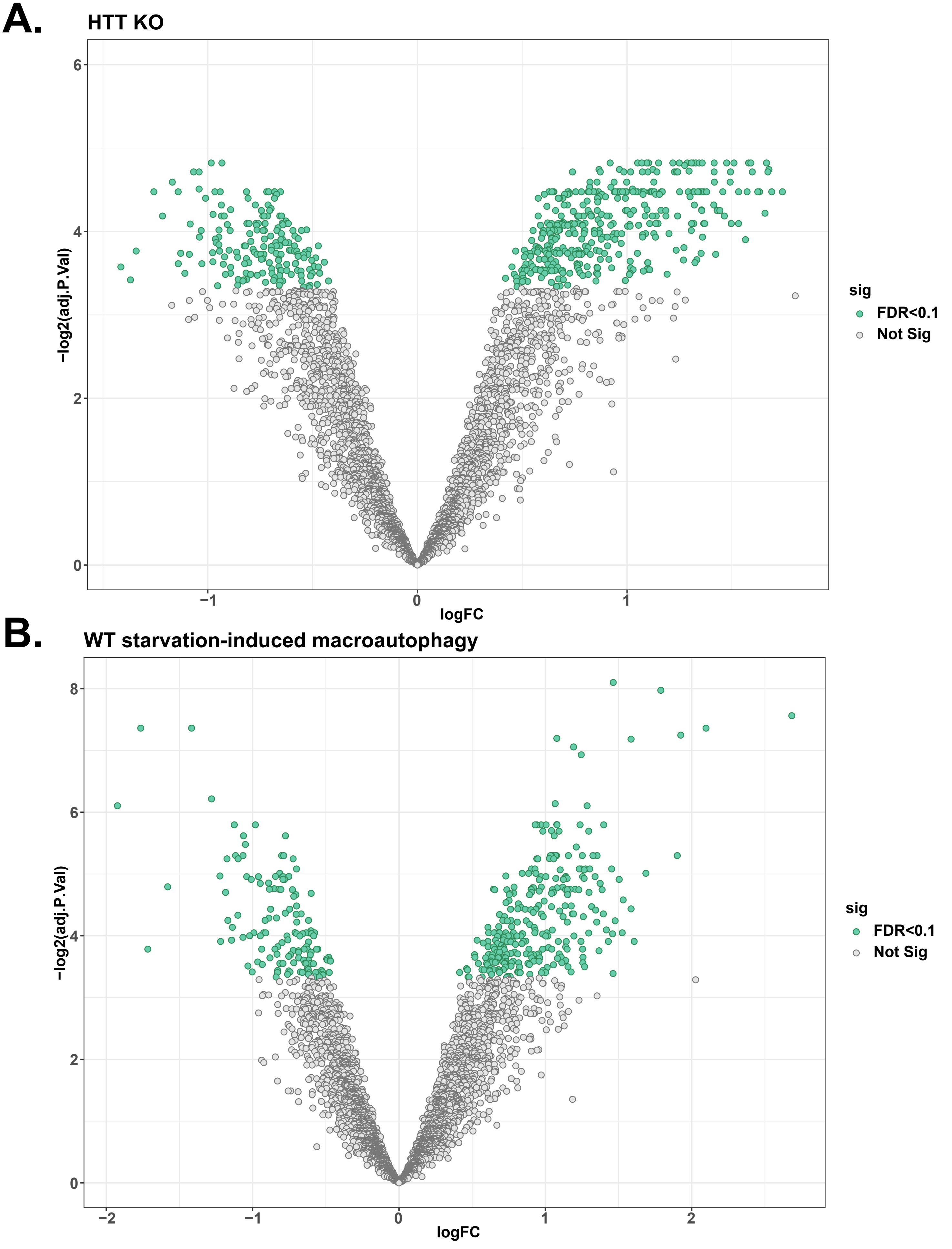

Fig. S3. Mass spec identifies differential protein abundance in HTT KO and WT starvation-induced macroautophagy lysosomal cargo. Volcano plots of log2(fold change) and negative log2(adjusted p value) values from differential proteins identified in our mass spec study for HTT KO (A) and starvation-induced macroautophagy (B). Statistical significance was determined by a false-discovery rate < 10%.

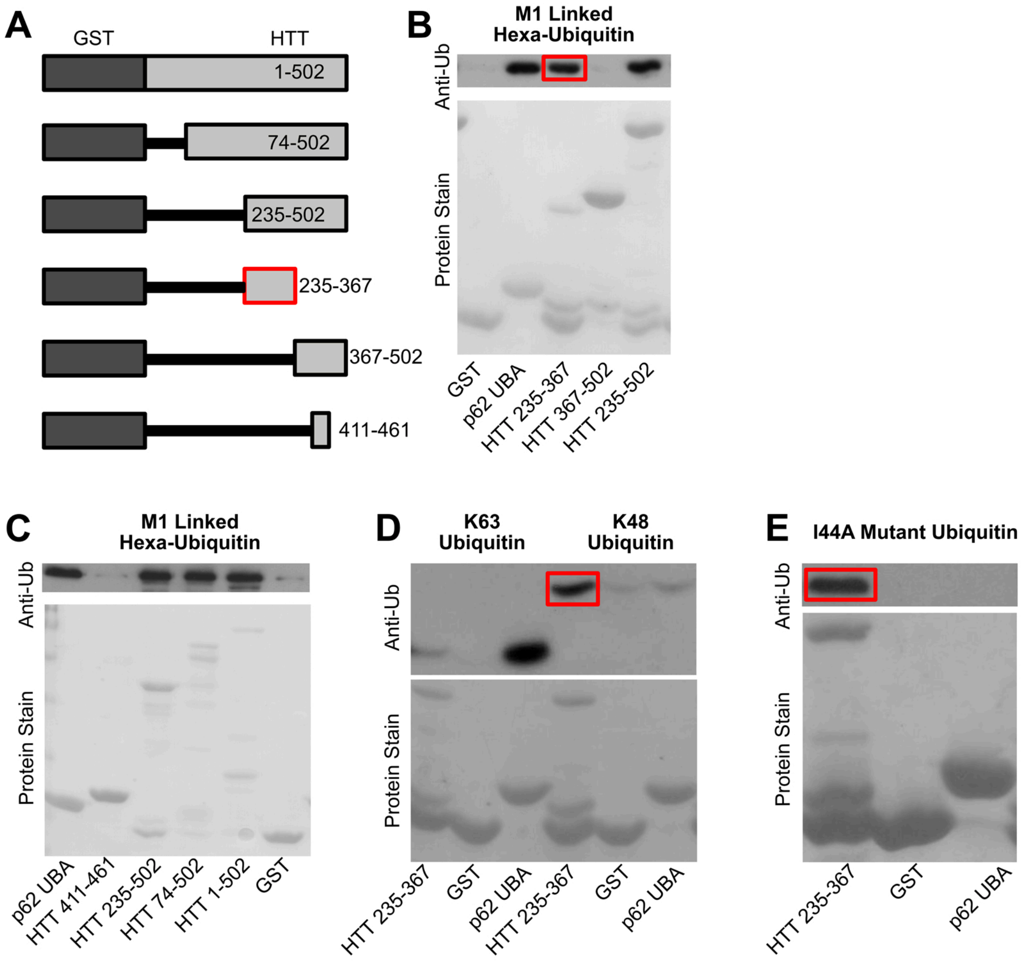

Fig. S4. N-terminal HTT fragments interact non-covalently with ubiquitin *in vitro*. GST pull-down assays were performed to identify interaction with ubiquitin of N-terminal HTT-GST fragments. (A) HTT fragments tested for *in vitro* interaction with ubiquitin. (B-C) Each HTT fragment was tested for interaction with ubiquitin chains. The ubiquitin binding domain of p62 fused to GST was used as a positive control, and GST alone was used as a negative control. Linear hexa-ubiquitin chains were used as bait. (D) K63-linked chains and K48-linked chains were used as bait for HTT fragments. (E) I44A mutant ubiquitin was tested for interaction with HTT fragments.

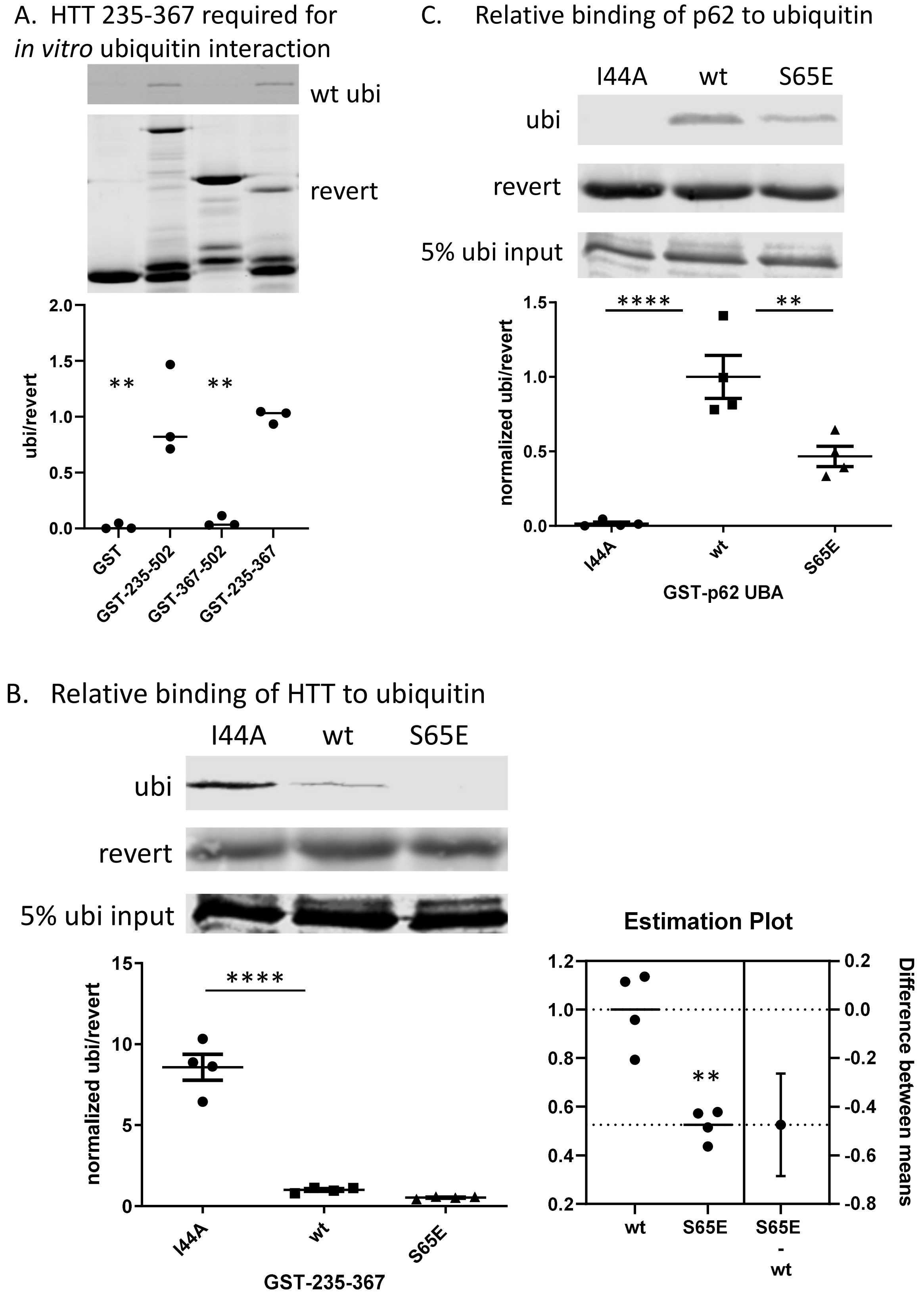

Fig. S5. ***In vitro*** **GST-pull down analysis demonstrates a direct interaction between HTT amino acids 235-367 and ubiquitin.** (A) Levels of wt linear ubiquitin bound to GST-HTT constructs vs. GST alone control were analyzed relative to the abundance of the full-length (top band) of the GST-fusion protein determined by revert protein stain. Western analysis with anti-ubiquitin MAB and revert whole protein stain were quantitated using a LI-COR imager. GST-367-502 and GST alone control had significantly reduced binding than GST-235-367, demonstrating it is essential for the *in vitro* interaction with wt ubiquitin. (B) HTT amino acids 235-367 interact more strongly with I44A than wt ubiquitin *in vitro* and GST-235-367 interacts more strongly with wt than S65E ubiquitin. (C) p62's UBA ubiquitin-binding domain interacts with wt ubiquitin more strongly than S65E, but does not interact with I44A ubiquitin. For B and C, ubiquitin western signals were calculated relative to the GST-HTT 235-367 or GST-p62 UBA revert protein stain, then normalized to the ubiquitin input level of I44A, wt, or S65E linear ubiquitin. Analysis was statistically analyzed using One-Way ANOVA with Bonferroni's multiple comparisons test or by unpaired t-test (wt vs. S65E for GST-HTT 235-367).

Mutations made within the HTT Ubiquitin-binding domain 235-367

A. 3 potential UBMs mutated:

```
  IKVLLKAFIANLKSSSPTIRRTAAGSAVSI   UBM1 HTT 250-279
YSWLLNVLLGLLVPVEDEHSTLL            UBM2 HTT 290-312
  ILGVLLTLRYLVPLLQQQVKDTSLKGSFGV   UBM3 HTT 313-342
```

UBM1: L261A K262A UBM2: V302A P303A UBM3: V324A P325A

B. 2 potential UIMs mutated:

```
KVLLKAFIANLKSSSPTIRR   UIM1
     A259   S263
TAAGSAVSICQHSRRTQYFY   UIM2
     I279   S283
```

UIM1: A259G S263A UIM2: I279G S283A

C. 1 potential DISUBM mutated:

Disordered Ubiquitin Binding Motif (DISUBM): XΩXXXΩX, where Ω is an aromatic residue, and X is any residue with a strong bias toward negatively charged residues

XXXXXX[F/Y/W]XXX[F/Y/W]XXXXXX

HSRRTQY FYSW LLNVLLGLLVPVEDE

288 292 305 306 307

DISUBM: Y288G F289G Y290G S291A W292G

D. All mutants with secondary structure:

235-367 ubiquitin-binding domain: potential UBM, UIM, DISUBM mutations

368-502 regulatory domain, mutations made within 368-502

Alpha Helices, Intrinsically Disordered Domains

```
235-
pkimasfgnfandneikvllkafianlksssptirrtaagsavsicqhsrrtqyfyswllnvllgl
lvpvedehstllilgvlltlrylvpllqqqvkdtslkgsfgvtrkemevspsaeqlvqvyeltlhh
tqhqdhnvvtgalellqqlfrtpppellqtltavggigqltaakeesggrsrsgsiveliagggss
cspvlsrkqkgkvllgeeealeddsesrsdvsssaltasvkdeisgelaassgvstpgsaghdiit
eqpr-502
```

UIM1: A259G S263A, UBM1: L261A K262A, UIM2: I279G S283A, DISUBM: Y288G F289G Y290G S291A W292G, UBM2: V302A P303A, UBM3: V324A P325A

R418A, S421A, S421D, S421A S434A, V423P, L425V, L425A, G428V, G429V, G430V, L437P, A452P

Fig. S6. Six potential ubiquitin-binding domains within HTT amino acids 235-367 were mutated and used for analysis in GST-pull down assays with I44A linear ubiquitin. Examination of HTT residues 235-367, required for the *in vitro* interaction of HTT with ubiquitin, revealed 3 possible ubiquitin-binding motifs (UBMs, A), 2 potential ubiquitin-interacting motifs (UIMs, B) and 1 potential disordered ubiquitin-binding motif (DisUBM, C (19)). The secondary structure of HTT's ubiquitin-binding domain UBD 235-367 and regulatory domain 368-502 showing alpha helices and intrinsically disordered regions (20, 21) involved in ubiquitin binding and HTT stability are shown in D, along with all the mutations described in this manuscript.

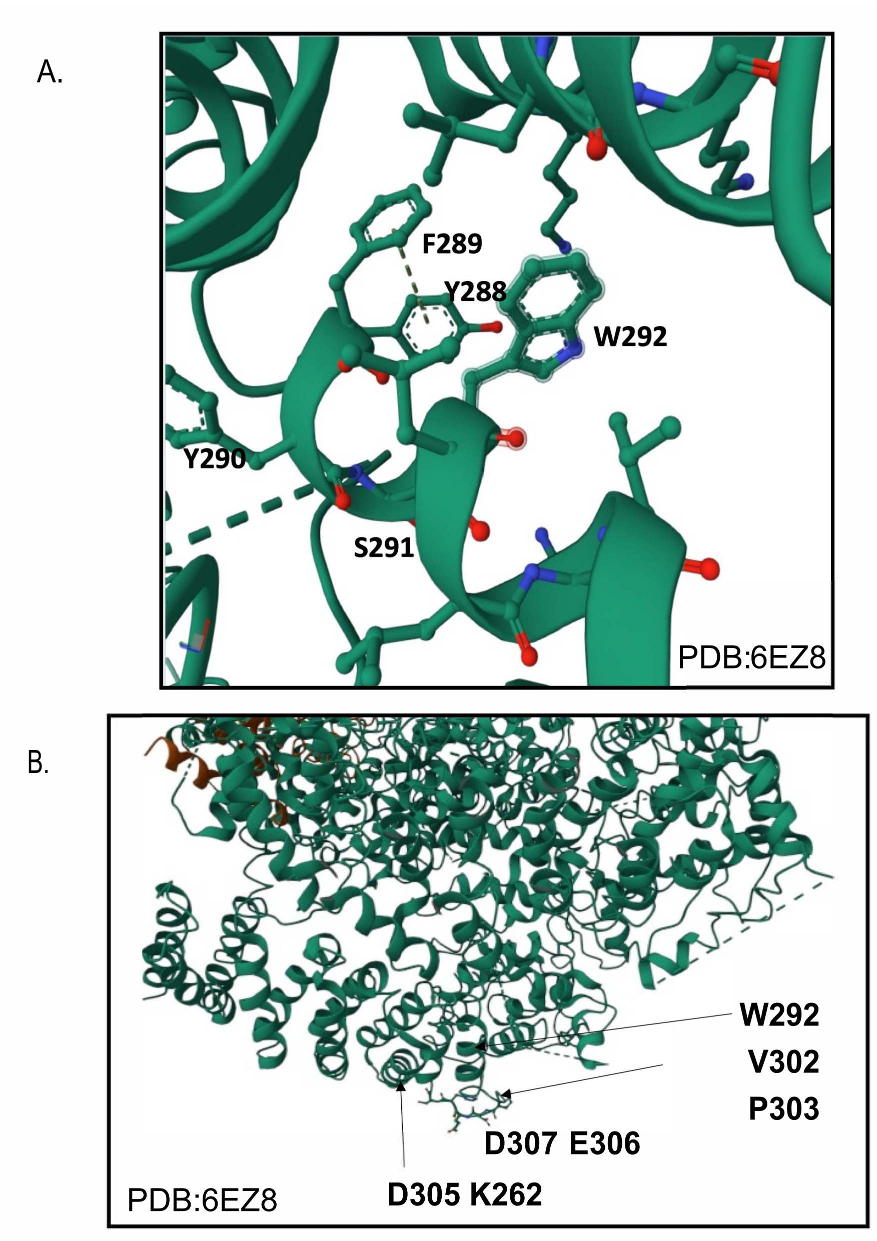

Fig. S7. Cryo-EM structure of HTT showing residues surrounding potential disordered ubiquitin-binding motif (DisUBM). PDB: 6EZ8 (20), the published cryo-EM structure of HTT, was used to depict residues found to be important for HTT's direct *in vitro* interaction with I44A ubiquitin through a potential DisUBM motif (19).

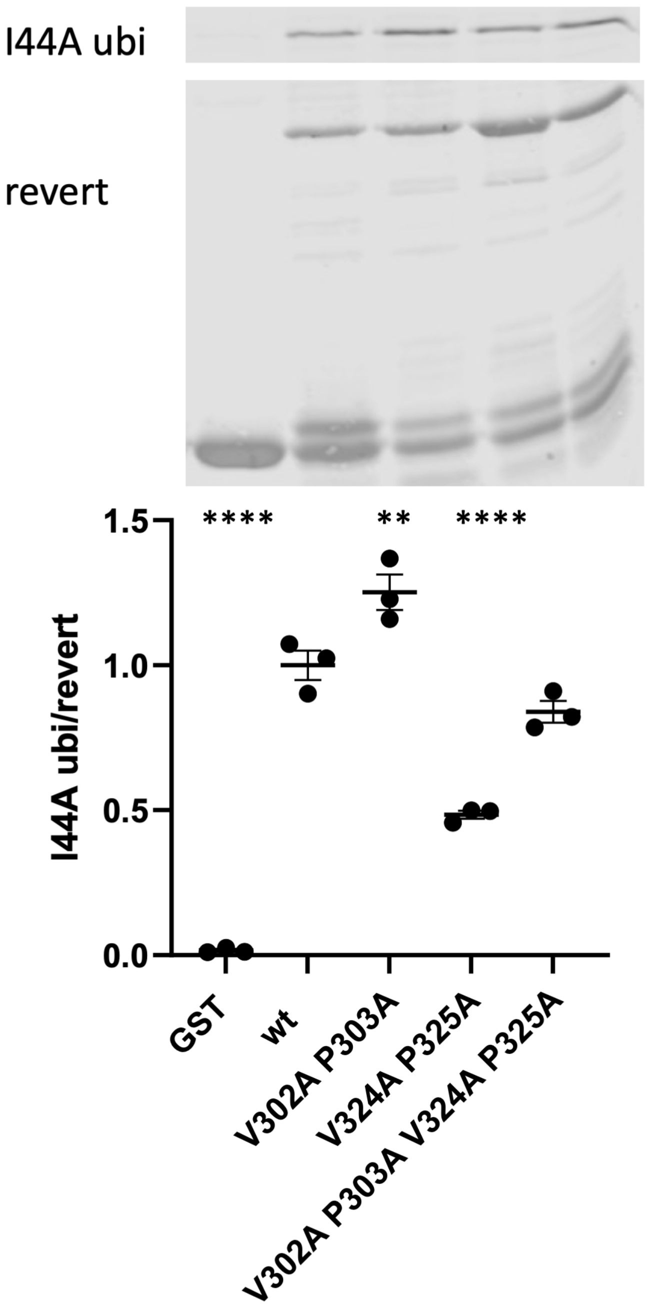

Fig. S8. Mutation of potential UBM2 (V302A P303A) enhances the *in vitro* interaction of GST-235-502 HTT fragment with I44A ubiquitin. GST pull-down analysis was used to evaluate the interaction of potential UBM2 (V302A P303A) and UBM3 (V324A P325A) and both mutations together (V302A P303A V324A P325A) *in vitro* with I44A ubiquitin. While mutation of UBM3 significantly reduced interaction with I44A ubiquitin compared with wt, mutation of UBM2 enhanced this interaction. The quadruple mutant had no significant effect. One-Way ANOVA with Bonferroni's multiple comparisons test was used for statistical analysis. Significance compared to wt shown.

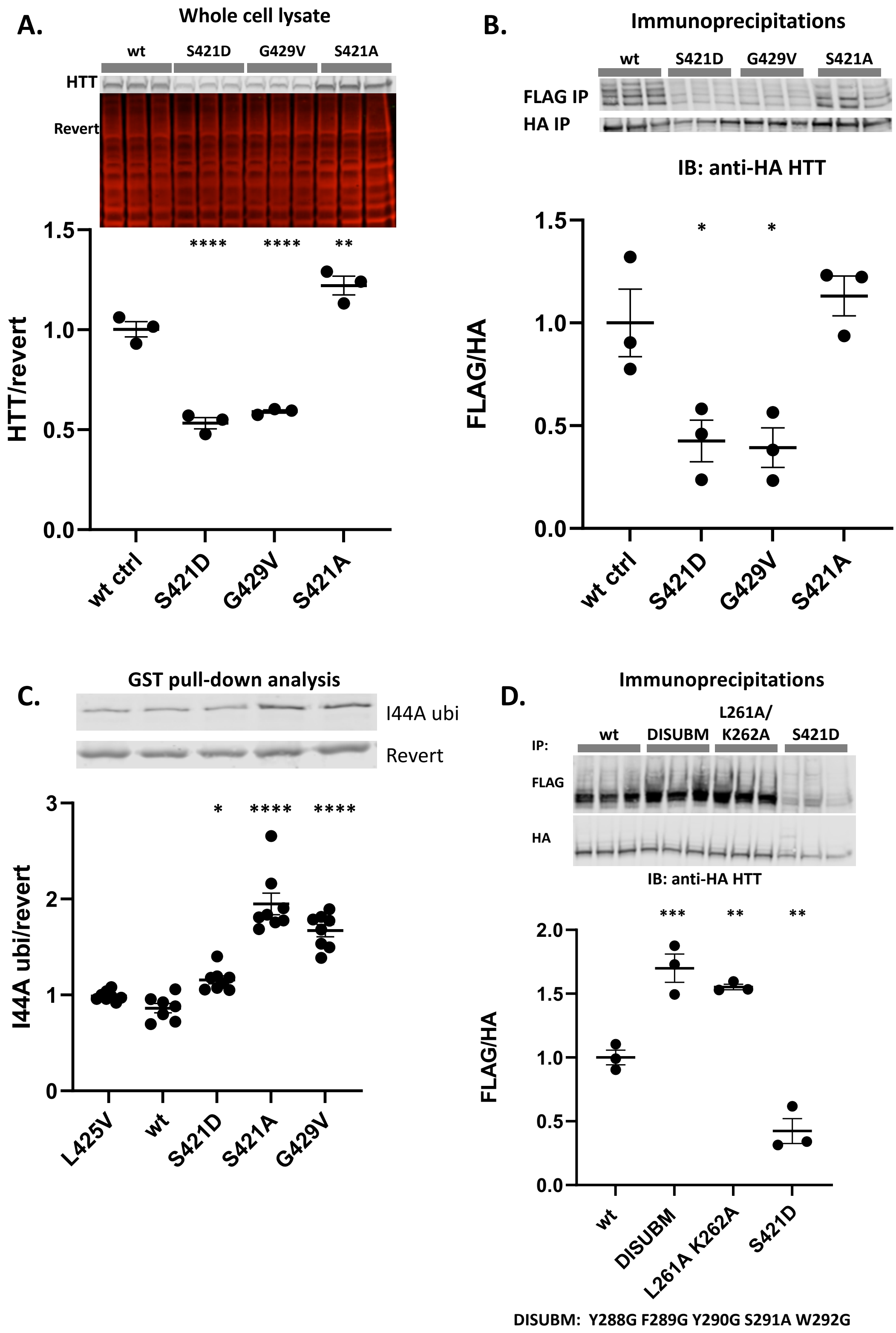

Fig. S9. HTT amino acids 367-502 impact HTT's stability and ubiquitin interaction. (A) Mimicking phosphorylation of HTT serine S421 (S421D) or G429V mutation within 17Q-1-502 HTT fragment both reduce HTT levels in whole St14A cell extract. Anti-HA antibody immunoreactivity of 17Q-1-502-HIS-HA-HA-HIS was normalized to protein loading (revert) in whole St14A cell lysate as analyzed with Empirica software using LI-COR. (B) 17Q-1-502 HTT-HIS-HA-HA-HIS was co-transfected with FLAG-ubiquitin, and St14A whole cell lysates were immunoprecipitated with either anti-HA (to pull down total HTT fragment) or anti-FLAG (to pull down ubiquitinated proteins). The level of HTT interaction with wt FLAG-ubiquitin was determined using a ratio of HTT pulled down in the anti-FLAG vs. the anti-HA immunoprecipitation. S421D and G429V both precipitated less well with ubiquitin, demonstrating a reduced level of interaction with wt ubiquitin in cells. (C). GST-pull down analysis using GST-HTT-235-502 fragment showed significantly enhanced in vitro I44A ubiquitin interaction of HTT with S421A and G421V HTT. (D) 17Q-1-502-HIS-HA-HA-HIS wt or mutant HTT fragment was co-expressed in St14A cells with FLAG-ubiquitin. Immunoprecipitation with anti-HA for total HTT was compared to HTT levels co-immunoprecipitated with FLAG-ubiquitin as detected on western with anti-HA antibody. DISUBM and UBM1 (L261A K262A) mutants had enhanced co-immunoprecipitation with wt ubiquitin, while S421D was reduced. A, B, C and D were all analyzed using One-Way ANOVA with Bonferroni's multiple comparisons test. Significance compared to wt shown.

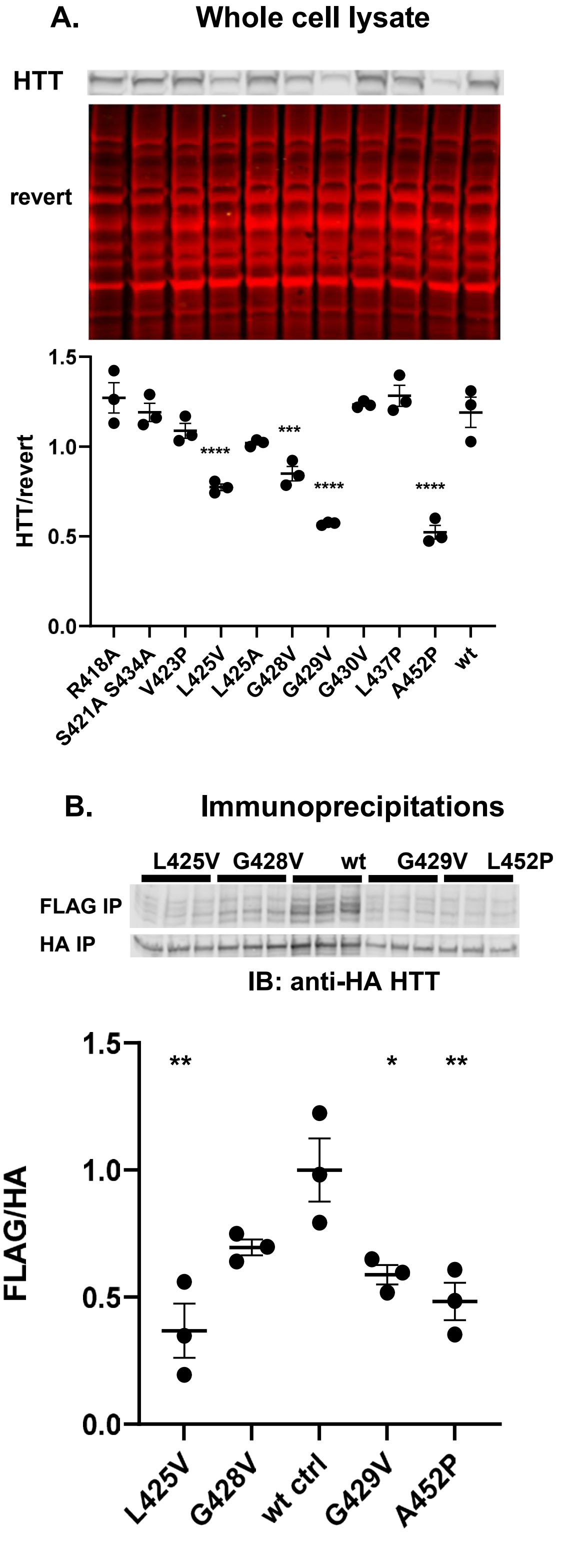

Fig. S10. Mutations within HTT 368-502 destabilize HTT fragment and reduce its co-immunoprecipitation with ubiquitin from St14A cells. (A) Four mutations were found to reduce abundance of 17Q-1-502-HIS-HA-HA-HIS fragments when expressed in St14A cells: L425V, G428V, G429V and A452P, as compared with wt. HTT levels were detected by anti-HTT antibody and normalized to total protein measured with revert protein stain, quantitated by Empirica software using LI-COR analysis. (B) 17Q-1-502-HIS-HA-HA-HIS HTT wt and mutant fragments were co-expressed with FLAG ubiquitin in St14A cells. Total HTT was pulled down with anti-HA monoclonal antibody, and separately ubiquitinated proteins were precipitated with anti-FLAG antibody. The relative co-immunoprecipitation of HTT with ubiquitin was quantitated comparing levels of anti-HA immunoreactivity coming down with anti-FLAG (FLAG-ubiquitinated proteins) vs. anti-HA (total HTT). The immunoblots were detected with rabbit polyclonal anti-HA antibody. 3 mutants, L425V, G429V, and A452P, were found to co-immunoprecipitate less efficiently with FLAG-wt ubiquitin from St14A cell extract, compared with wt. A and B were analyzed using One-Way ANOVA with Bonferroni's multiple comparisons test. Significance compared to wt shown.

Legends for Datasets S1 to S4

Dataset S1: Lyso-IP significant proteins comparing WT basal to WT HBSS
Dataset S2: Lyso-IP significant proteins comparing WT basal to HTT KO basal
Dataset S3: Pathway analysis of ubiquitinated and ubiquitin-associated proteins found to tandem co-purify with HTT 17Q-502 amino acid fragment
Dataset S4: List of 205 ubiquitinated and ubiquitin-associated proteins found to tandem co-purify with HTT 17Q-502 amino acid fragment, and their overlap with published proteomic databases.

SI References

1. V. V. Eapen, S. Swarup, M. J. Hoyer, J. A. Paulo, J. W. Harper, Quantitative proteomics reveals the selectivity of ubiquitin-binding autophagy receptors in the turnover of damaged lysosomes by lysophagy. *Elife* **10** (2021).
2. A. Ruzo *et al.*, Chromosomal instability during neurogenesis in Huntington's disease. *Development* **145** (2018).
3. M. E. Ehrlich *et al.*, ST14A cells have properties of a medium-size spiny neuron. *Exp Neurol* **167**, 215-226 (2001).
4. M. Abu-Remaileh *et al.*, Lysosomal metabolomics reveals V-ATPase- and mTOR-dependent regulation of amino acid efflux from lysosomes. *Science* **358**, 807-813 (2017).
5. A. Ordureau *et al.*, Global Landscape and Dynamics of Parkin and USP30-Dependent Ubiquitylomes in iNeurons during Mitophagic Signaling. *Mol Cell* **77**, 1124-1142 e1110 (2020).
6. E. L. Huttlin *et al.*, A tissue-specific atlas of mouse protein phosphorylation and expression. *Cell* **143**, 1174-1189 (2010).
7. K. C. Yang, S. M. Gorski, Protocol for analysis of RNA-sequencing and proteome profiling data for subgroup identification and comparison. *STAR Protoc* **3**, 101283 (2022).
8. H. Patel *et al.*, Proteomic blood profiling in mild, severe and critical COVID-19 patients. *Sci Rep* **11**, 6357 (2021).
9. G. D'Angelo *et al.*, Statistical Models for the Analysis of Isobaric Tags Multiplexed Quantitative Proteomics. *J Proteome Res* **16**, 3124-3136 (2017).
10. M. P. van Ooijen *et al.*, Identification of differentially expressed peptides in high-throughput proteomics data. *Brief Bioinform* **19**, 971-981 (2018).
11. N. Alsabeeh, B. Chausse, P. A. Kakimoto, A. J. Kowaltowski, O. Shirihai, Cell culture models of fatty acid overload: Problems and solutions. *Biochim Biophys Acta Mol Cell Biol Lipids* **1863**, 143-151 (2018).
12. D. L. Brasaemle, N. E. Wolins, Isolation of Lipid Droplets from Cells by Density Gradient Centrifugation. *Curr Protoc Cell Biol* **72**, 3 15 11-13 15 13 (2016).
13. B. Qiu, M. C. Simon, BODIPY 493/503 Staining of Neutral Lipid Droplets for Microscopy and Quantification by Flow Cytometry. *Bio Protoc* **6** (2016).
14. C. Yu *et al.*, Characterization of Dynamic UbR-Proteasome Subcomplexes by In vivo Cross-linking (X) Assisted Bimolecular Tandem Affinity Purification (XBAP) and Label-free Quantitation. *Mol Cell Proteomics* **15**, 2279-2292 (2016).
15. J. Cox *et al.*, A practical guide to the MaxQuant computational platform for SILAC-based quantitative proteomics. *Nat Protoc* **4**, 698-705 (2009).
16. M. L. Seibenhener *et al.*, Sequestosome 1/p62 is a polyubiquitin chain binding protein involved in ubiquitin proteasome degradation. *Mol Cell Biol* **24**, 8055-8068 (2004).
17. C. Smith-Geater *et al.*, Aberrant Development Corrected in Adult-Onset Huntington's Disease iPSC-Derived Neuronal Cultures via WNT Signaling Modulation. *Stem Cell Reports* **14**, 406-419 (2020).
18. R. C. Laker *et al.*, A novel MitoTimer reporter gene for mitochondrial content, structure, stress, and damage in vivo. *J Biol Chem* **289**, 12005-12015 (2014).
19. J. E. Dreier *et al.*, A context-dependent and disordered ubiquitin-binding motif. *Cell Mol Life Sci* **79**, 484 (2022).
20. Q. Guo *et al.*, The cryo-electron microscopy structure of huntingtin. *Nature* **555**, 117-120 (2018).
21. R. J. Harding *et al.*, Design and characterization of mutant and wildtype huntingtin proteins produced from a toolkit of scalable eukaryotic expression systems. *J Biol Chem* **294**, 6986-7001 (2019).
